# DRAM1 plays a tumor suppressor role in NSCLC cells by promoting lysosomal degradation of EGFR

**DOI:** 10.1038/s41419-020-02979-9

**Published:** 2020-09-17

**Authors:** Ji Geng, Rong Zhang, Xiao Yuan, Haidong Xu, Zhou Zhu, Xinxin Wang, Yan Wang, Guoqiang Xu, Wenjie Guo, Junchao Wu, Zheng-Hong Qin

**Affiliations:** 1 Department of Pharmacology and Laboratory of Aging and Nervous Diseases, Jiangsu Key Laboratory of Neuropsychiatric Diseases, College of Pharmaceutical Sciences, Soochow University, Suzhou, 215123 China; 2 Pathology Department, The First Affiliated Hospital of Soochow University, Suzhou, 215123 PR China; 3 Mr. and Mrs. Ko Chi Ming Centre for Parkinson’s Disease Research, School of Chinese Medicine, Hong Kong Baptist University, Hong Kong, SAR China; 4 State Key Laboratory of Pharmaceutical Biotechnology, School of Life Sciences, Nanjing University, 163 Xianlin Avenue, Nanjing, 210093 PR China; 5 Department of Pathology, Stanford University School of Medicine, Stanford, CA USA

**Keywords:** Lysosomes, Oncogenesis

## Abstract

Lung cancer is the leading cause of cancer-associated mortality worldwide. DNA damage-regulated autophagy modulator 1 (DRAM1) plays an important roles in autophagy and tumor progression. However, the mechanisms by which DRAM1 inhibits tumor growth are not fully understood. Here, we report that DRAM1 was decreased in nonsmall-cell lung carcinoma (NSCLC) and was associated with poor prognosis. We confirmed that DRAM1 inhibited the growth, migration, and invasion of NSCLC cells in vitro. Furthermore, overexpression of DRAM1 suppressed xenografted NSCLC tumors in vivo. DRAM1 increased EGFR endocytosis and lysosomal degradation, downregulating EGFR signaling pathway. On one side, DRAM1 interacted with EPS15 to promote EGFR endocytosis, as evidence by the results of proximity labeling followed by proteomics; on the other, DRAM1 recruited V-ATP6V1 subunit to lysosomes, thereby increasing the assemble of the V-ATPase complex, resulting in decreased lysosomal pH and increased activation of lysosomal proteases. These two actions of DRAM1 results in acceleration of EGFR degradation. In summary, these in vitro and in vivo studies uncover a novel mechanism through which DRAM1 suppresses oncogenic properties of NSCLC by regulating EGFR trafficking and degradation and highlights the potential value of DRAM1 as a prognostic biomarker in lung cancers.

## Introduction

Lung cancer is the most common cause of cancer death globally, and nonsmall-cell lung cancer (NSCLC) accounts for the most lung cancer cases^1^. Activated mutations have been identified in multiple oncogenes, including EGFR, ERBB2/3, ALK, KRAS, ROS, MET, AKT, and BRAF in NSCLC^2^. Aberrant EGFR or EGFR-activating mutations activate various downstream signaling pathways to promote the proliferation, migration, and invasion of NSCLC. Treatment with third-generation EGFR-tyrosine kinase inhibitors (TKIs) has markedly improved overall response rate (~79%) and progression-free survival (~64%) in EGFR-mutant NSCLC patients^3^, while long-term chemotherapy induces mutations of EGFR T790M and C797S, conferring the inevitable acquired resistance and frequent de novo resistance of NSCLC patients to TKIs^4^. The mutations of EGFR in NSCLC are concentrated in exons 18–20, encoding the tyrosine kinase domain, and they affect the ATP binding pocket of the tyrosine kinase domain and lead to ligand-independent activation of EGFR. Under a steady state or EGF stimulation, the internalized mutant EGFR travels via the canonical endosome-lysosome route for degradation^5^.

DNA damage-regulated autophagy modulator 1 (DRAM1) encodes a six transmembrane protein mainly located in lysosomes to induce autophagy and is downregulated in multiple human cancers^6^. DRAM1 directs newly synthesized amino acid transporters to lysosomes and drives lysosomal amino acid efflux^7^. In addition to lysosomes, DRAM1 isoforms are also partly localized to peroxisomes, autophagosomes, and the endoplasmic reticulum^8^. The reported mechanisms of DRAM1-triggered autophagy are mainly focused on promoting the generation of autophagy and increasing the fusion between autophagosomes and lysosomes. DRAM1 takes part in p62-dependent selective autophagy to engulf the entire mycobacteria to defend against mycobacterial infection^9^. In acute myocardial infarction (AMI), DRAM1 exerted cardiomyocyte protection by increasing ATG7 expression and interacting with ATG7; additionally, DRAM1 enhances the conversion of autophagosomes to autophagolysosomes^10,11^. However, the mechanism by which DRAM1 promotes the fusion between autophagosomes and lysosomes has not been completely elucidated.

We have previously demonstrated that DRAM1 augmented lysosomal acidification and promoted the fusion of lysosomes with autophagosomes^12^. DRAM1 also recruited Bax to lysosomes and caused the release of cathepsin B to initiate Bid-mediated apoptosis of tumor cells under stress conditions^13^. We also demonstrated that DRAM1 regulated the activation of the IGF-1 receptor and inhibited the downstream PI3K–AKT–mTOR pathway in the presence of growth factors, resulting in autophagy activation and decreased cell proliferation in several human cancer cells^14^. Moreover, we found that DRAM1 was involved in maintaining normal organization of the Golgi apparatus, suggesting that DRAM1 plays a role in Golgi functions^15^. It was recently reported that acidification of mycobacterium marinum (Mm)-containing vesicles was strongly reduced in DRAM1 mutants in tuberculosis (TB) model^16^. In the current study, we found that DRAM1 was decreased in NSCLC and negatively correlated with EGFR levels. In addition, overexpression of DRAM1 inhibited the proliferation, migration, invasion, and EMT of NSCLC cell lines harboring mutant EGFR in vitro and in vivo. We demonstrated that DRAM1 promoted EGFR endocytosis through interacting with EPS15, and lysosomal degradation of EGFR via recruiting V-ATP6V1 subunit to lysosomes. Hence, our findings uncover a novel role of DRAM1 in EGFR endocytic trafficking and lysosomal degradation, making this protein a potential prognostic marker and/or therapeutic target for NSCLC patients.

## Materials and methods

### Gene overexpression and knockdown

Cells were bought from Cell Bank of Shanghai Institute of Cell Biology. HEK293T cells and A549 cells were cultured in DMEM with 10% fetal bovine serum. NCI-H1975 cells and PC9 cells were cultured in RPMI-1640 containing 10% fetal bovine serum. To overexpress or reduce DRAM1, lentiviral mCherry vector-mediated and lentiviral GFP vector-mediated DRAM1-FLAG overexpression constructs, lentiviral mCherry vector-mediated DRAM1 shRNAs (Lv-NC: TTCTCCGAACGTGTCACGT; sh-DRAM1: CCTACAGTCCATCATCTCTTA) and EPS15 shRNA and EPS15 siRNA (sh/si-EPS15: GCCCAGAATGGATTGGAAGTTTC) were synthesized by Genepharm (Shanghai, China). A total of 5 × 10^5^ H1975 cells or PC9 cells were plated in 6-well plates in medium with 10% FBS and 2 μg/ml polybrene and infected with 50 μl of virus. The medium was refreshed, and puromycin (2 μg/ml) was added for selection after 24 h. After 48 h of transfection, the expression of mCherry or GFP was detected with fluorescence microscopy.

### Construction of plasmids and transfection

The 3FLAG-DRAM1-GCaMP6 expression plasmid, TMEM192-3HA, and DRAM1-BioID2-FLAG were generated by homologous recombination. 3FLAG-DRAM1, DRAM1, and TMEM192-3HA were generated by PCR subcloning of the human DRAM1 and TMEM192 coding sequences, GCaMP6 and BioID2 were generated by PCR subcloning of GCaMP6 plasmid and BioID2 plasmid, respectively. 3FLAG-DRAM1 and GCaMP6 or DRAM1 and BioID2-FLAG were homologously recombined using the Hieff Clone® Plus Multi One Step Cloning Kit (Yeason, China) according to the manufacturer’s instructions. Information on labeling organelle with plasmids is included in Supplementary Table 1.

HEK293T, A549 cells, H1975 cells, or PC9 cells were cultured at 70–80% confluence in 24-well plates on the day before transfection. Lipofectamine 2000 reagent (0.5 μl) was diluted with 25 μl Opti-MEM and gently blended with 25 μl Opti-MEM containing 0.5 μg plasmid. The mixture was placed at room temperature for 15 min and added to the cell culture medium. The medium was changed to fresh medium after 6 h, and immunofluorescence assay was conducted after another 42 h.

### Western blot assay

The tumor tissues and cells were harvested and lysed in RIPA lysis buffer for 30 min on ice, the proteins were then quantified and Western blotting was performed as previously described^17^. All information on antibodies used in the study is included in Supplementary Table 2.

### Co-IP

Proteins of H1975 cells, DRAM1-overexpressing H1975 cells, A549 cells, and DRAM1-overexpressing A549 cells were extracted using cold RIPA lysis buffer followed by centrifugation at 14,000 × *g* for 15 min at 4 °C. A volume of 0.5 mg/ml protein was added to 20 μl Anti-FLAG agarose beads (A2220, Sigma-Aldrich), and they were slowly shaken on a rotating shaker at 4 °C overnight. After centrifugation 500 × *g* for 3 min, the pellet was washed with precooled PBS three times, and the beads were boiled in 2× loading buffer (Beyotime, China). Then, the supernatants were collected and subjected to Western blot analysis for EPS15, ATP6V1D, and ATP6V0D, respectively.

### RT-qPCR

Quantitative real-time PCR was performed using SYBR Green PCR master mix (Takara, RR420A) in a total volume of 20 µl on 7500 Real-Time PCR System (Applied Biosystems) as follows: 95 °C for 30 s, 40 cycles of 95 °C for 5 s, and 60 °C for 30 s. All results were normalized to the expression of α-Tubulin, and relative quantification was calculated by the 2^−ΔΔCt^ method.

### Immunohistochemistry

Sections were deparaffinized, rehydrated through graded ethanol, and washed in PBS. Antigen retrieval was performed in citrate buffer for 10 min followed by blocking endogenous peroxidase in 3% H_2_O_2_ for 15 min. Sections were blocked with 5% BSA and incubated with antibody for 3 h at room temperature. After washing in 1× PBS, the slides were processed with a GTVisinTM antimouse/antirabbit immunohistochemical analysis kit (GeneTech, China) according to the manufacturer’s instructions.

### Cell proliferation assay

H1975 control cells, DRAM1-overexpressing H1975 cells, PC9 control cells, DRAM1-overexpressing PC9 cells, PC9-negative cells, and DRAM1-knockdown PC9 cells were seeded into 96-well plates at a density of 3000 cells per well. After 24, 48, 72, and 96 h, 10 μl of CCK-8 was added into each well to measure cell proliferation.

### Immunofluorescence

Cells were washed three times with PBS, fixed with 4% paraformaldehyde for 30 min followed by 0.5% Triton X-100 for 10 min, incubated with 3% bovine serum albumin for 1 h at room temperature, and then incubated with primary antibodies overnight. After being washed three times with 0.01 M PBS for 5 min, cells were incubated with secondary antibodies labeled with Alexa Fluor™ 488/555/633 (1:1000, Proteintech, China) for 1 h at room temperature. Cells were washed three times with 0.01 M PBS for 3 min. Nuclei were stained with DAPI (10 µg/ml) for 5 min at room temperature. Images were acquired on an LSM-710 confocal microscope (Zeiss, Germany) using a 60× objective.

### Immunopurification of lysosomes (LysoIP)

Lysosomes were isolated as previously described^18^. Cells in five 10 cm plates were quickly rinsed twice with PBS and then scraped using PBS (136 mM KCl, 10 mM KH_2_PO_4_, pH 7.25) and centrifuged at 800 × *g* for 5 min at 4 °C. Pelleted cells were gently homogenized with a 1 ml Dounce homogenizer. The homogenate was then centrifuged at 800 × *g* for 5 min at 4 °C, and the supernatant containing lysosomes was incubated with 50 μl PBS-prewashed anti-HA magnetic beads (HY-K0201, MedChemExpress, China) on a gentle rotator shaker for 20 min. The tube was placed on a magnetic separator, and the supernatant was removed. Beads were washed 2 times using 1 ml PBS and mixed with 50 μl 2× loading buffer and heated in boiling water for 5 min. The mixture was vortexed and centrifuged at 12,000 × *g* for 15 min at 4 °C, and the supernatant was collected for Western blot analysis.

### Lysosomal pH assay

LysoSensor Green DND-189 (40767ES50, Yeason, China) and DQ-Green BSA (D-12050, Molecular Probe) were used to detect lysosomal pH. LysoSensor Green DND-189 accumulates in acidic environments to exhibit more fluorescence and is often used to measure the pH of lysosomes. The proteolysis of DQ-Green BSA in acidic compartments results in fragment formation with fluorescence dequenching, which is observed as an increase in fluorescence intensity. Cells were stained with 1 μM Lysotracker Green DND-189 diluted in RPMI-1640 at room temperature for 30 min or 10 μg/ml DQ-Green BSA for 6 h, washed twice with PBS and collected using a scraper. Flow cytometry using a FACSCalibur (BD) and FlowJo software were performed to detect and analyze the fluorescence intensity.

### BioID2-mediated proximity-tagging system

As described previously with slight modification^19^, the BioID2-mediated proximity-tagging system and affinity purification (AP)-MS approach were performed to identify DRAM1-interacting proteins. Briefly, 5 × 10^7^ HEK293T cells transiently expressing DRAM1-BioID2 were stimulated with 30 μM biotin for 24 h, and the cells were collected and lysed in 2 ml of RIPA buffer for 30 min on ice. After centrifugation at 12,000 × *g* for 15 min, supernatant was collected and incubated with 50 μl avidin resin (V2012, Promega) at 4 °C overnight. Beads were collected by centrifuging at 500 × *g* for 5 min and washed twice with RIPA lysis. Finally, beads were resuspended in 100 μl elution buffer (50 mM Tris-HCl, pH 8.0, 150 mM NaCl, 5 mM EDTA, 0.5 mM Biotin) in a shaker for 10 min and centrifuged at 500 × *g* for 5 min. The supernatants were collected and boiled in loading buffer followed by Western blot analysis.

For liquid chromatography-mass spectrometry (LC-MS), the collected elution buffer was reduced with 5 mM Tris (2-carboxyethyl)phosphine (TCEP) for 30 min at 37 °C and alkylated with 10 mM iodoacetamide, followed by digestion with trypsin at 37 °C overnight. Formic acid was added to the peptide solution before LC-MS/MS.

### EGFR degradation and recycling

There is rapid endocytosis at physiological temperature (37 °C), whereas at 4 °C, ligand-receptor internalization is inhibited without affecting binding. To measure EGFR endosomal-lysosomal trafficking and degradation, A549 cells, DRAM1-overexpressing A549 cells, PC9 cells and DRAM1-overexpressing PC9 cells were serum-starved overnight and stimulated with 100 ng/ml EGF (10605-HNAY, Sinobiological, China) for 0 min, 15 min, 30, 60, or 180 min. Subsequently, the cells were harvested and subjected to SDS-PAGE and Western blotting to detect EGFR protein levels and its downstream pathway activity. For fluorescence-based EGFR trafficking and degradation, A549 cells, DRAM1-overexpressing A549 cells, PC9 cells and DRAM1-overexpressing PC9 cells were serum-starved overnight, incubated with 100 ng/ml Alexa Fluor 488-conjugated EGF (E13345, Invitrogen) for 30 min at 4 °C, washed twice with PBS, and shifted to 37 °C for 0, 15, 30, 60, or 180 min. After fixation, fluorescence images were taken using an LSM-710 confocal microscope (Zeiss, Germany), and fluorescence intensities were measured by flow cytometry (FACS Calibur, BD).

The EGFR recycling assay was conducted as described^20^. To measure the amount of total internalized EGFR, cells were incubated with serum-free medium overnight and treated with 100 ng/ml Alexa Fluor 488-conjugated EGF for 30 min at 4 °C, washed twice with PBS and fixed with 4% paraformaldehyde. To measure the recycling of internalized EGFR, serum-starved cells were stimulated with 100 ng/ml EGF for 30 min at 4 °C to bind EGFR completely (pulse), washed and chased for 0, 30, or 60 min to allow EGFR recycling. Cells were then treated with Alexa Fluor 488-conjugated EGF for 30 min at 4 °C, washed and fixed as described above.

### Subcutaneous tumor growth assay

Five-week-old male nude BALB/c mice were obtained from Soochow University. Mice were housed in SPF conditions with food and water ad libitum, a 12 h light/dark cycle and controlled (22–23 °C) temperature. Animal welfare and experimental procedures were carried out strictly in accordance with the Guide for the Care and Use of Laboratory Animals (National Institutes of Health, USA) and the related ethical regulations of Soochow University. Mice were randomly injected subcutaneously with 5 × 10^5^ PC9 or 5 × 10^5^ DRAM1-overexpressing PC9 cells in 30 μl of RPMI-1640 on the right back flank. Two weeks later, tumor size was measured with electronic calipers every 4 days through blind outcome assessment. Tumor volumes (V) were calculated by the formula *V* = (*X*^2^*Y*)/2, where *X* and *Y* are the shortest and longest diameters of the tumor, respectively. Mice were sacrificed on day 41 after tumor cell injection. For determination of a role of DRAM1 in gefitinib chemotherapy, mice bearing PC9 cells or DRAM1-overexpressing PC9 cells were orally administered gefitinib (S1025, Selleck), dissolved in 0.5% CMC-Na, at dose of 50 mg/kg/day for 13 days. Tumor size and weight were measured. Tumors were collected and fixed with 4% formaldehyde or stored at −80 °C.

### Tumor specimens from patients

Surgical resections of lung cancer tissues were collected from first diagnosed patients (50–60-year-old male) without chemoradiotherapy at The First Affiliated Hospital of Soochow University from 2017 to 2018 according to approval from the Ethics Committee of Soochow University. Written informed consent was obtained from all patients prior to enrollment. Human lung cancer tissue array (IWLT-N-70L43) was purchased from Wuhan Iwill Biological Technology Co., China.

### Bioinformatic analyses

The Oncomine database (http://www.oncomine.org) is a cancer microarray database. We adopted the Oncomine database to further validate the expression of DRAM1 in lung cancer. The differential expression analysis was directly performed using Oncomine online analysis tools. DRAM1 expression between squamous lung cancer and paired normal tissue from five patients was analyzed using data extracted from the Gene Expression Omnibus (GEO) with accession number GSE3268. Overall survival data were obtained from Kaplan–Meier Plotter (http://kmplot.com/analysis/) for lung cancer. MS-identified proteins were analyzed for KEGG pathways and biological processes (BP) to determine whether these proteins had overlapping functions using the Database for Annotation, Visualization, and Integrated Discovery (DAVID)^21^.

### Statistical analysis

Data were analyzed using the statistical software GraphPad Prism (Version 7.00 for Windows, Graph Pad Software, CA, USA) and are expressed as mean ± standard deviation (SD). Statistical significance was evaluated by one-way analysis of variance (ANOVA) followed by Tukey’s multiple comparison test (multiple groups) or the paired or unpaired *t*-test (two groups). All experiments were successfully replicated three times, and a value of *P* < 0.05 was considered statistically significant.

## Results

### Lower levels of DRAM1 is associated with poor clinical outcomes in lung cancers

**Fig. 1:**
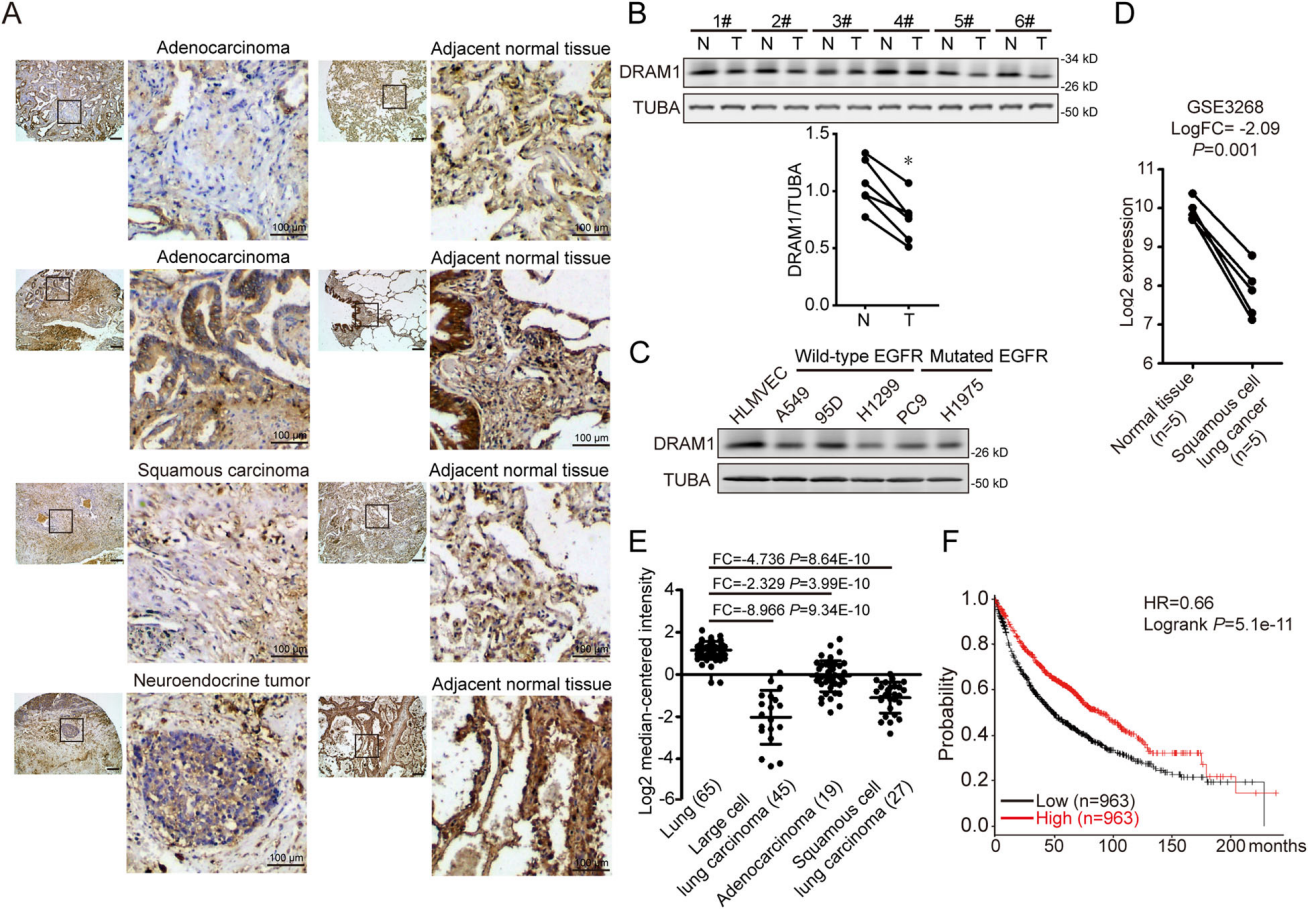
Decrease in DRAM1 is significantly associated with lung cancer progression. **a** DRAM1 expression in human lung cancer tissues and paired normal tissues. Scale bar in overview is 200 μm and in a detailed view of selected area is 100 μm. **b** DRAM1 and EGFR expression in six pairs of adenocarcinomas and matched adjacent nontumor specimens from patients. T: adenocarcinomas; N: adjacent nontumor tissues. *P < 0.05, paired *t*-test. **c** Western blot analysis of DRAM1 levels in noncancerous lung cells (HLMVEC) and NSCLC cell lines (A549, 95D, H1299, PC9, and H1975). **d** DRAM1 mRNA levels in squamous cell lung cancers and adjacent normal tissues from the GEO dataset (GSE3268). **e** DRAM1 mRNA levels in normal lung tissues and different types of lung cancers from the Oncomine database. FC fold change. **f** The correlation between low DRAM1 expression and poor prognosis in lung cancer patients from the Kaplan–Meier Plotter database.

To determine the clinical significance of DRAM1 in lung cancer, immunohistochemical staining was utilized to measure the expression of DRAM1 in NSCLC patient specimens and in matched normal tissues. Representative immunohistochemical data of adenocarcinoma, squamous cell carcinoma, neuroendocrine tumor, and matched normal tissues are shown in Fig. 1a. Compared with normal lung tissues, the intensity of DRAM1 immunoreactivity was decreased in tumor tissues. Western blot results also showed that DRAM1 protein levels in NSCLC tissues were also lower than that in paired normal tissues **(**Fig. 1b**)**. Moreover, DRAM1 protein in NSCLC cells (EGFR-wild-type NSCLC cell lines such as A549, 95D and NCI-H1299 cells; and EGFR-mutant NSCLC cell lines such as PC9 and NCI-H1975 cells.) was lower than that in human lung microvascular endothelial cells (HLMVEC) (Fig. 1c). GEO database and Oncomine database were utilized to analyze the differential expression of DRAM1 in normal lung and tumor tissues. As shown in Fig. 1d, e, lower DRAM1 mRNA expression was detected in NSCLC samples than in matched normal lung tissues. Additionally, the Kaplan–Meier survival plot clearly revealed that low DRAM1 mRNA level was associated with a poor prognosis for the overall survival time of lung cancer patients (Fig. 1f).

Taken together, these results suggest that DRAM1 is negatively associated with poor outcomes in lung cancer progression.

### DRAM1 disrupts oncogenic transformation of NSCLC cells in vivo and in vitro, and increases the sensitivity of EGFR–TKI

**Fig. 2:**
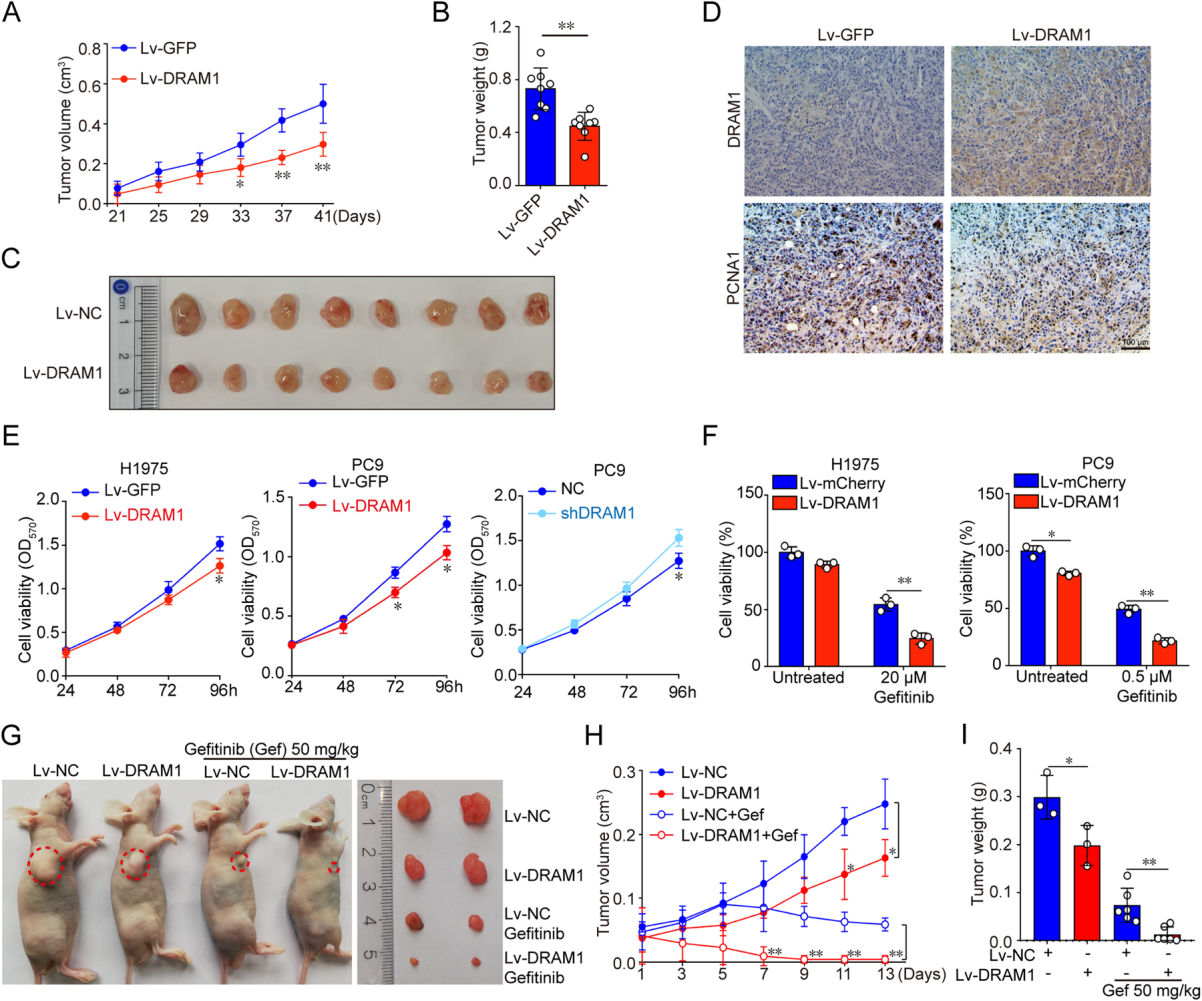
DRAM1 inhibits NSCLC cell growth in vivo and in vitro, and increases the sensitivity of NSCLC to EGFR–TKI in vitro and in vivo. **a** PC9 cells and DRAM1-overexpressing PC9 cells were subcutaneously injected into the flank of male BALB/c nude mice (*n* = 8). The tumor volumes were measured and monitored every 4 days. **b** The weights of subcutaneous PC9 and DRAM1-overexpressing PC9 tumors on the 41st day after injection. **c** Nude mice were sacrificed, and representative images of tumors dissected from each group of mice were taken. **d** Representative images of immunohistochemical (IHC) staining for DRAM1 and PCNA1 in PC9 xenograft tumor tissues and DRAM1-overexpressing PC9 tumor tissues. Scale bar: 100 µm. **e** Growth curves of DRAM1-overexpressing and DRAM1-knockdown NSCLC cells. **f** H1975 and H1975-DRAM1 cells were treated with 20 μM gefitinib for 48 h, and cell viability was monitored with CCK-8 assay. PC9 and PC9-DRAM1 cells were treated with 0.5 μM gefitinib for 48 h, and cell viability was monitored with CCK-8 assay. To detect whether DRAM1 sensitizes to EGFR-TKI in vivo, BALB/c nude mice bearing PC9 cells and DRAM1-overexpressing PC9 cells were orally administrated with 50 mg/kg gefitinib once a day for 13 days. **g** Representative images of tumor-bearing mice and tumors in different groups. **h** Tumor volume curve and **i** tumor weight were measured. Data are shown as the mean ± SD of three independent experiments. *P < 0.05, **P < 0.01.

To test the role of DRAM1 in NSCLC progression, subcutaneous transplantation model using PC9 cells, an EGFR-mutant (exon 19 deletion) NSCLC cell line with a TKI-sensitizing mutation, and DRAM1-overexpressing PC9 cells in nude mice were established. Compared with control tumors, overexpression of DRAM1 inhibited PC9 tumor growth with a significant reduction in tumor growth rate, tumor size and weight (Fig. 2a–c). PCNA1 staining further showed a significant decrease in the number of proliferating cells in DRAM1-overexpressing tumors compared with the control group (Fig. 2d). In addition, DRAM1 was overexpressed or knocked down using lentiviral transfection in NCI-H1975 cells, an EGFR-mutant (T790M) NSCLC cell line with the EGFR TKI-acquired resistance mutation, and in PC9 cells. Proliferation was assessed by CCK-8 assay and colony formation assay, and results in Figs. 2e and SI 2A showed that overexpression of DRAM1 led to a significant decrease in cell proliferation. Conversely, DRAM1 silencing increased cell proliferation.

DRAM1-overexpressing cells exhibited a cobblestone morphology, reflecting EMT morphological changes, and DRAM1-silenced cells had an elongated mesenchymal shape (Fig. SI 1A–C). EMT is closely correlated with metastasis and invasion of cancer cells. Therefore, the effects of DRAM1 on cell migration and invasion were further examined using wound healing assay and transwell assay. DRAM1 overexpression decreased wound closure and penetrated cells, suggesting that DRAM1 inhibited migration and invasion of NSCLC cells (Fig. SI 2B–D). Furthermore, DRAM1 upregulation decreased EMT markers including α-SMA and N-cadherin in NSCLC cells (Fig. SI 2E). Meanwhile, DRAM1 sensitized EGFR-mutant NSCLC cell line H1975 and PC9 cells to gefitinib, an EGFR-targeting TKI (Fig. 2f). DRAM1 also increased the tumor inhibition of gefitinib in vivo. As shown in Fig. 2g–i, the decreased tumor volume and tumor weight were significant between DRAM1-Gefitinib and Gefitinib alone groups than that between DRAM1–PC9 and PC9 groups, suggesting that DRAM1 overexpression enhanced gefitinib sensitivity.

Taken together, these results suggest that DRAM1 significantly inhibits the oncogenetic potential of NSCLC cells in vivo and in vitro, including the inhibition of growth, EMT, and increasing TKI sensitivity in vitro and in vivo.

### DRAM1 decreases EGFR and EGFR signaling in NSCLC cells

**Fig. 3:**
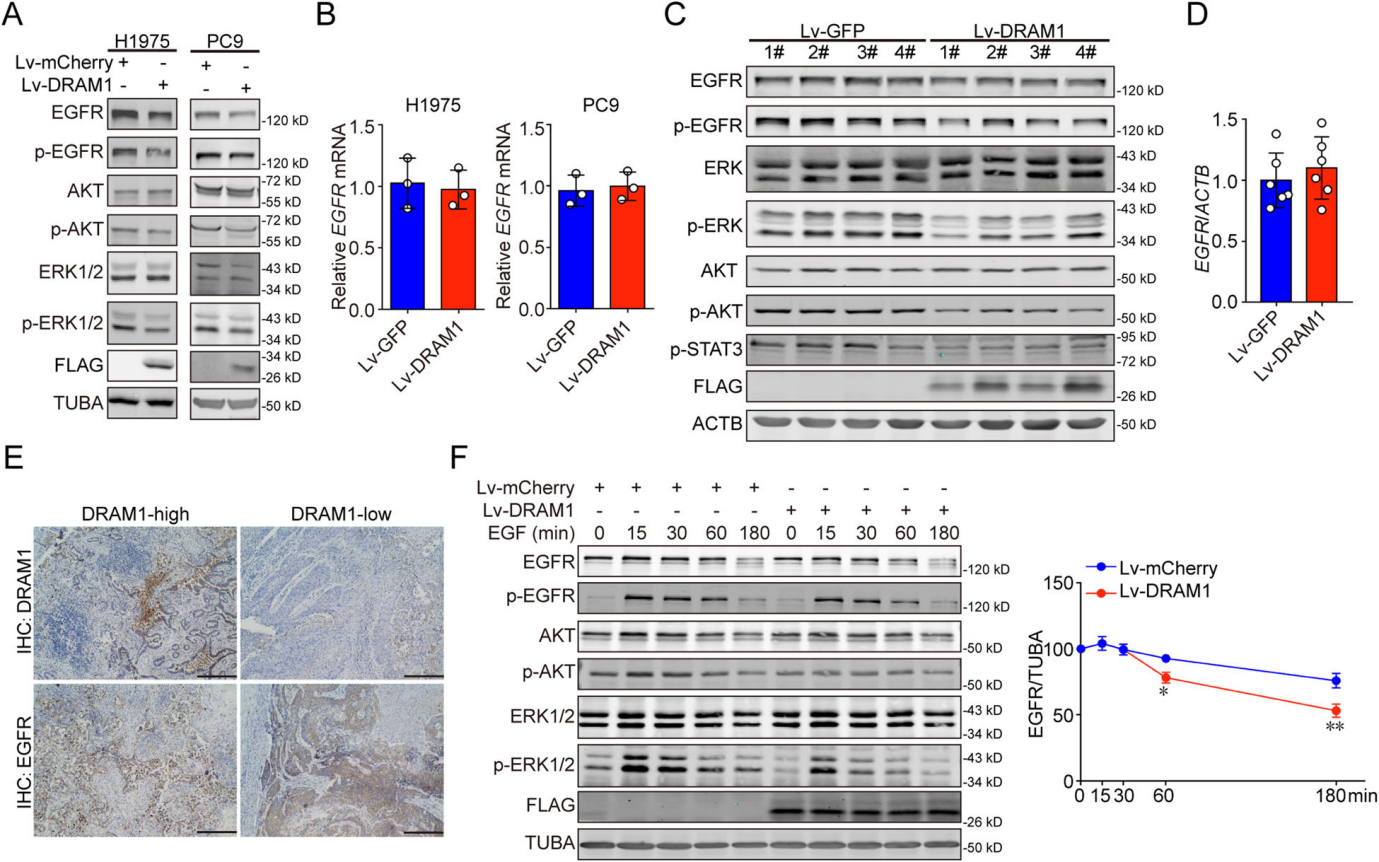
DRAM1 decreases EGFR expression and its signaling pathway in vitro and in vivo. **a** The protein levels of EGFR, p-EGFR (Tyr1068), AKT, p-AKT (Ser473), ERK1/2, and p-ERK1/2 (Thr202/Tyr204) in H1975, DRAM1-overexpressing H1975, PC9, and DRAM1-overexpressing PC9 cells were measured using Western blot analysis. **b** The mRNA levels of EGFR in the indicated cells were detected using RT-qPCR. **c** The protein levels of EGFR, p-EGFR (Tyr1068), ERK1/2, p-ERK1/2 (Thr202/Tyr204), AKT, p-AKT (Ser473), and p-STAT3 (Tyr705) in PC9 xenograft tumors and DRAM1-overexpressing PC9 xenograft tumors were determined using Western blot analysis. **d** The levels of EGFR mRNA in control tumor tissues and DRAM1-overexpressing tumor tissues was detected using RT-qPCR. **e** DRAM1 and EGFR expression in lung cancer tissues were detected with immunohistochemistry. Scale bar is 200 μm. **f** A549 cells and A549-DRAM1 cells were serum-starved overnight and treated with 100 ng/ml EGF for different duration of times. The levels of EGFR, p-EGFR (Tyr1068), AKT, p-AKT (Ser473), ERK1/2, and p-ERK1/2 (Thr202/Tyr204) were measured using Western blot analysis. The quantification was performed by densitometry. Data are presented as the mean ± SD of three independent experiments. *P < 0.05, **P < 0.01.

To further investigate the mechanism of DRAM1 in tumor suppressive activity, EGFR, p-EGFR, p-AKT, and p-ERK proteins were detected in DRAM1-overexpressing NSCLC cells. As shown in Figs. 3a, b and SI 3A, DRAM1 overexpression decreased EGFR protein and downregulated the levels of p-EGFR, p-AKT and p-ERK but had no significant effect on EGFR mRNA in H1975 and PC9 cells. Assessment of xenografted NSCLC tumors also revealed that overexpression of DRAM1 resulted in lower protein levels of EGFR, p-EGFR, p-ERK, p-AKT, and p-STAT3 than that in PC9 cells with no effect on EGFR mRNA, consistent with the in vitro results mentioned above (Fig. 3c, d and SI 3B). These results indicate that DRAM1 decreases EGFR protein levels. Sections of lung cancer tissues were stained with specific DRAM1 and EGFR antibodies to verify the correlation between DRAM1 and EGFR, and specimens with high DRAM1 staining were negatively correlated with EGFR staining (Fig. 3e and SI 3C). EGFR secretion through exosomes has been reported^22^, and CD63, ALIX, TSG101, and SDCBP are key proteins involved in the generation of exosomes. No significant changes of these proteins were observed between control cells and DRAM1-overexpressing cells (Fig. SI 3D). To determine whether the overexpression of DRAM1 promoted EGFR endocytosis and degradation in lung cancer cells, A549 cells containing wild-type EGFR were used to measure EGFR degradation in the presence of EGF. As described in the Fig. 3f, upregulation of DRAM1 increased the rate of EGFR degradation and decreased EGFR downstream protein levels of p-AKT and p-ERK. Similar results were also observed in DRAM1-deficient cells, where the remaining EGFR protein in DRAM1-knockdown cells was much higher than in control cells (Fig. SI 3E).

Collectively, these findings indicate that DRAM1 promotes the clearance of EGFR in NSCLC cells.

### DRAM1 accelerates endocytic trafficking and degradation of EGFR

Upon EGF binding, activated wild-type EGFR is rapidly internalized to endosomes/lysosomes for degradation or recycled back to the cell surface. The trafficking route of EGFR was detected in A549 cells and DRAM1-overexpressing A549 cells by comparing the endocytic EGFR. In DRAM1-knockdown cells, the colocalizations of EEA1-positive early endosomes and Texas Red-EGF were reduced after 15 min stimulation, suggesting that the pathway from the cellular surface to early endosomes was downregulated (Fig. SI 4A). In addition, the colocalizations of lysosomes and Texas Red-EGF were also decreased in DRAM1-deficient cells than control cells after a 120 min chase, suggesting that the pathway from early endosomes to lysosomes was impaired. The fluorescence of Texas Red-EGF in Fig. SI 4B also showed that, in contrast to control cells, more EGF-positive vesicles were still observed after 120 min EGF stimulation, indicating that EGFR endocytosis from surface, endosomes to lysosomes is interrupted in DRAM1-knockdown cells. Moreover, the faster colocalization of Alexa-488 labeled EGF to early endosomes (RAB5 positive) and late endosomes (RAB7 positive) in DRAM1-overexpressing cells at 10 and 30 min also suggested that DRAM1 accelerated EGF trafficking (Fig. SI 5A, B).

**Fig. 4:**
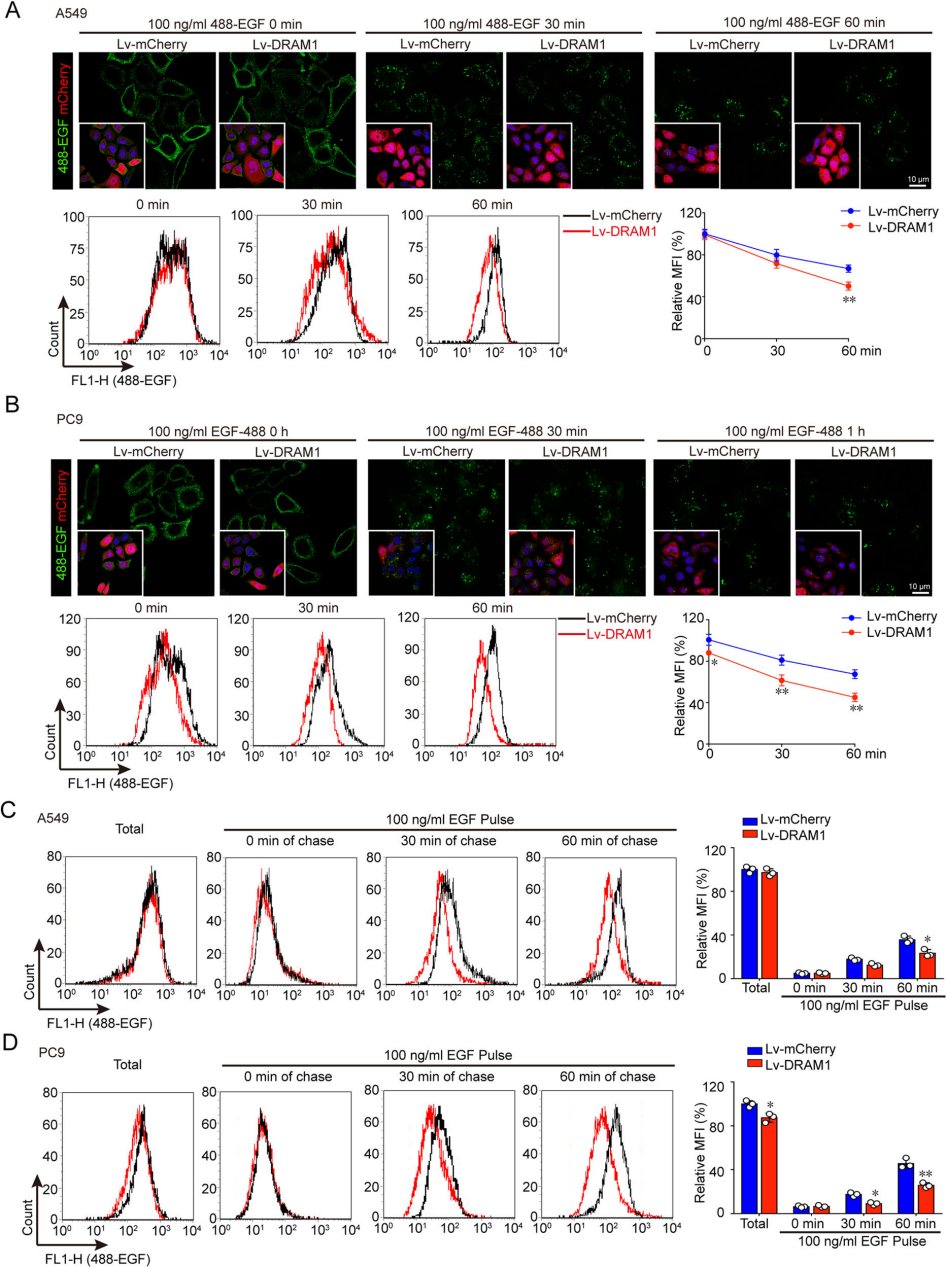
DRAM1 accelerates the degradation of EGFR and decreases EGFR recycling in NSCLC cells. **a**, **b** A549 cells and A549-DRAM1 cells or PC9 cells and PC9-DRAM1 cells were serum-starved overnight and stimulated with 100 ng/ml of Alexa Fluor 488-EGF at 4 °C for 30 min, followed by additional different duration of times at 37 °C. The fluorescence pictures were captured, and the intensities were measured by a FACS-Calibur. Scale bar: 10 μm. **c**, **d** A549 cells and A549-DRAM1 cells or PC9 cells and PC9-DRAM1 cells were cultured in serum-free medium. Total represents the surface EGFR binding Alexa Fluor 488-EGF. For the EGFR recycling assay, cells were stimulated with 100 ng/ml EGF for 30 min at 4 °C (pulse) followed by 0, 30, or 60 min of chase at 37 °C. The cells were then incubated with 100 ng/ml Alexa Fluor 488-EGF for 30 min at 4 °C, washed and detected using flow cytometry. Data are presented as the mean ± SD of three independent experiments. *P < 0.05, **P < 0.01.

In DRAM1-overexpressing NSCLC cells, DRAM1 decreased fluorescent EGF intensity, and the sharper slope of the decrease in fluorescence intensity of 488-EGF, compared with NSCLC cells, also suggested that DRAM1 accelerated the clearance of EGFR in NSCLC cells (Fig. 4a, b). EGFR recycling was further measured, the ratio of surface EGFR in DRAM1-overexpressing NSCLC cells to control cells was significantly decreased after chasing for 60 min, suggesting that recycled EGFR was reduced in DRAM1-overexpressing cells (Fig. 4c, d).

### DRAM1 interacts with EPS15 to accelerate EGFR endocytosis in NSCLC cells

It has been reported that DRAM1 is predominantly located in lysosomes and late endosomes^8^. As shown in Fig. SI 6A–H, we confirmed that DRAM1 mainly localized to lysosomes (mCherry-LAMP1 positive), and later endosomes (GFP-RAB7 positive and GFP-RAB9 positive) in lung cancer cell. Lysosomes expressing 3FLAG-DRAM1 were isolated with antiFLAG agarose beads, illustrating that the N-terminus of DRAM1 oriented to the cytosol (Fig. SI 6I). In addition, lysosomal Ca^2+^ release induced by ML-SA1 increased fluorescent intensity of DRAM1–GCaMP6, indicating the C-terminus of DRAM1 oriented to the cytosol (Fig. SI 6J, K). DRAM1–GFP colocalized with LysoTracker Red and maintained GFP fluorescence (Fig. SI 6L), indicating that the C-terminus of DRAM1 faced to the cytosol. These findings suggest that DRAM1 with both N-terminals and C-terminals facing the cytosol was mainly located in lysosomes.

**Fig. 5:**
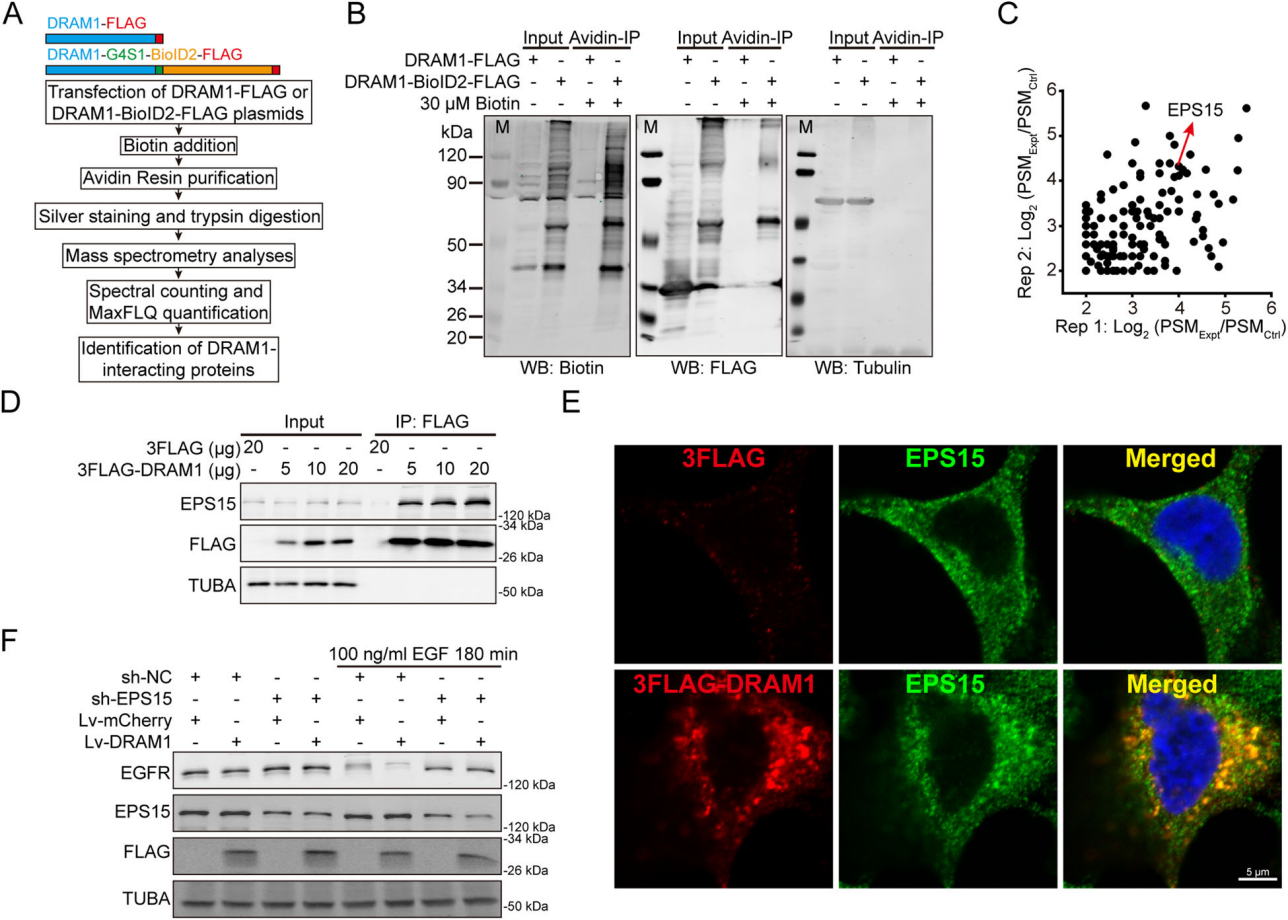
DRAM1 interacts with EPS15 to promote EGFR endocytosis. **a** Flowchart for the affinity purification and LC-MS/MS identification of DRAM1-interacting proteins through BioID2-mediated biotinylation. **b** HEK293T cells were transiently transfected with DRAM1 and DRAM1-BioID2 plasmid for 24 h and treated with 30 μM biotin for another 24 h, and biotinylated proteins were isolated with avidin resin. Biotinylated proteins, FLAG and tubulin were analyzed with Western blot assay. **c** Scatter plot for the log2 ratio (log2 > 2) of biotinylated proteins of the control (Ctrl) and experimental (Expt) samples obtained from two independent experiments. **d** A549 cells were transfected with different concentrations of 3FLAG–DRAM1 plasmid, and co-IP was performed with anti-EPS15 antibody. **e** A549 cells were transfected with 3FLAG–DRAM1, and an immunofluorescence assay was carried out using antiFLAG and antiEPS15 antibodies to investigate the colocalization of these two proteins. Scale bar: 5 μm. **f** A549 cells and A549–DRAM1 cells were transfected with control shRNA and shEPS15. Cells were serum-starved overnight and stimulated with 100 ng/ml EGF for 180 min, EGFR were detected using Western blot analysis.

BioID2, a biotin ligase-catalyzed proximity label, can label biotin to proximate proteins^23^. BioID2 was fused to the C-terminus of DRAM1 and expressed in HEK293T cells. Many DRAM1-interacting proteins were biotinylated after treatment with 30 μM biotin. These biotinylated proteins were pulled down and identified using MS analysis (Fig. 5a, b). The identified proteins (Fig. SI 7A) are listed in Supplementary Table 3. EPS15 was one of the proteins above the threshold (log2 > 2) (Fig. 5c). These proteins were analyzed by KEGG pathways and biological processes (BP) using DAVID, indicating that DRAM1 regulates vesicle-mediated transport (Fig. SI 7B, C). We found that exogenously expressed DRAM1 interacted with EPS15 using co-IP assay (Fig. 5d). The overlay image on confocal microscopy revealed partial colocalization of exogenous DRAM1 and EPS15 (Fig. 5e). To detect whether EPS15 was involved in EGFR trafficking in DRAM1 overexpressing cells, EPS15 was knocked down in DRAM1 overexpressing A549 cells and EGF-induced EGFR endocytic degradation was analyzed. As shown in Fig. 5f, EPS15 knockdown resulted in delayed EGFR endocytic degradation in A549 cells and diminished the enhancement of EGFR degradation in DRAM1-overexpressing cells. Meanwhile, decreasing EPS15 also diminished the inhibitory effects of DRAM1 overexpressing on cell growth, migration, and invasion in PC9 cells (Fig. SI 10).

Taken together, these results indicate that DRAM1 promotes EGFR endocytosis via interacting with EPS15 and is EPS15-dependent.

### DRAM1 increases lysosomal acidification through assembling lysosomal V-ATPase complex

**Fig. 6:**
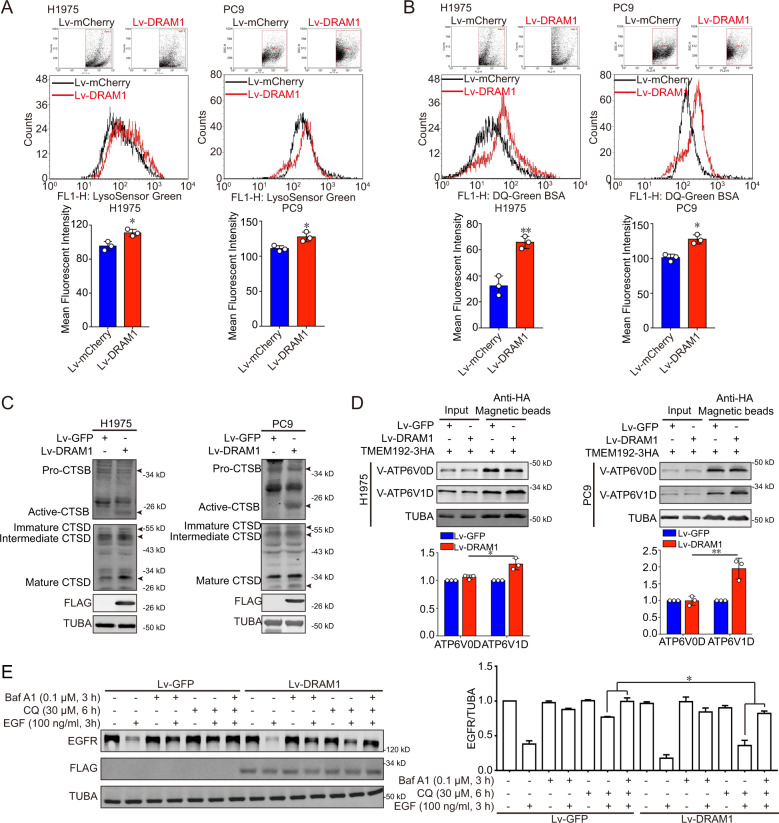
DRAM1 decreases lysosomal pH to degrade EGFR in NSCLC cells. **a** Cells were loaded with 1 μM LysoSensor Green DND-189 for 40 min to detect intravesicular pH, and positive DRAM1-overexpressing cells were gated to analyze LysoSensor Green using flow cytometry. **b** Cells were loaded with 10 μg/ml DQ-Green BSA for 6 h to detect intravesicular pH, and positive DRAM1-overexpressing cells were gated to analyze DQ-Green BSA using flow cytometry. **c** Western blot analysis of activated cathepsin B (CTSB) and mature cathepsin D (CTSD) in H1975 cells, H1975-DRAM1 cells, PC9 cells, and PC9–DRAM1 cells. Arrows indicated different forms of protein. **d** H1975 cells, H1975-DRAM1 cells, PC9 cells, and PC9–DRAM1 cells were transfected with TMEM192-3HA plasmid, and lysosomes were isolated with LysoIP. The protein levels of V-ATP6V0D and V-ATP6V1D in lysosomes were measured by Western blot analysis, and quantification was performed by densitometry. **e** A549 cells and A549-DRAM1 cells were starved overnight along with 0.1 μM bafilomycin A1 (Baf A1) for 3 h or 30 μM chloroquine (CQ) for 6 h, then cells were stimulated with 100 ng/ml EGF for 3 h, the EGFR protein was measured using Western blot analysis, and quantification was performed by densitometry. Data are presented as the mean ± SD of three independent experiments. *P < 0.05, **P < 0.01.

The role of DRAM1 on lysosomal functions including lysosome number and lysosomal acidification were measured. The intensity of the fluorescence emission from lysosomes showed no significant difference between DRAM1-overexpressing cells and control cells (Fig. SI 8A). Staining with LysoSensor Green DND-189, a pH indicator that exhibits a pH-dependent increase in fluorescence intensity upon acidification, showed that lysosomal acidification measured by flow cytometry was markedly augmented in DRAM1-overexpressing H1975 and PC9 cells compared to that in control cells (Fig. 6a). A similar phenomenon was observed using DQ-Green BSA, another pH indicator of lysosomal acidification (Fig. 6b). Cathepsin B proenzyme and immature cathepsin D can be processed into active cathepsin B and mature cathepsin D in the acidic milieu of lysosomes, which is dependent on lysosomal acidification. In contrast to control cells, DRAM1 increased the contents of active cathepsin B and mature cathepsin D (Fig. 6c). Meanwhile, active cathepsin B and mature cathepsin D were diminished in DRAM1-knockdown cells (Fig. SI 8B).

Together, these data suggest that DRAM1 increased lysosomal acidification and protease activation in NSCLC cells.

Our previous research reported that downregulation of DRAM1 inhibited lysosomal V-ATPase activity^12^. V-ATPase is composed of peripheral V1 and integral membrane V0 subcomplexes and is distributed in the plasma membrane and endosomal/lysosomal compartments. DRAM1 had no direct interaction with V-ATP6V1D or V-ATP6V0D, the subunit of the V1 or V0 subcomplex, respectively using co-IP assay (Fig. SI 9A). To investigate whether DRAM1 promoted the assembly of the lysosomal V-ATPase supercomplex, lysosomes were isolated using LysoIP. As presented in Fig. 6d, DRAM1 significantly promoted localization of peripheral V-ATP6V1D to lysosomes, without affecting the localization of V-ATP6V0D. Immunofluorescence further confirmed that DRAM1 obviously increased the colocalization between mCherry-LAMP1-labeled lysosomes and V-ATP6V1D in NSCLC cells (Fig. SI 9B–E).

To elucidate whether DRAM1-induced lysosomal acidification contributes to EGFR trafficking and degradation, bafilomycin A1 (Baf A1), an inhibitor of V-ATPase, and chloroquine (CQ), an inhibitor of lysosomal acidification, were applied to A549 cells and DRAM1-overexpressing A549 cells before EGF stimulation. As depicted in Fig. 6e, DRAM1-drived enhancement of EGFR degradation was blocked by Baf A1 and CQ, and Baf A1 exhibited greater inhibition of EGFR degradation than CQ did in DRAM1-overexpressing cells. Baf A1, at concentrations that have no effect on growth, migration and invasion of PC9 cells, promoted cell growth, migration and invasion in DRAM1 overexpressing PC9 cells (Fig. SI 10A–D).

Therefore, the above results indicate that DRAM1 facilitates EGFR lysosomal degradation through V-ATPase-mediated lysosomal acidification, suppressing EGFR signaling.

## Discussion

**Fig. 7:**
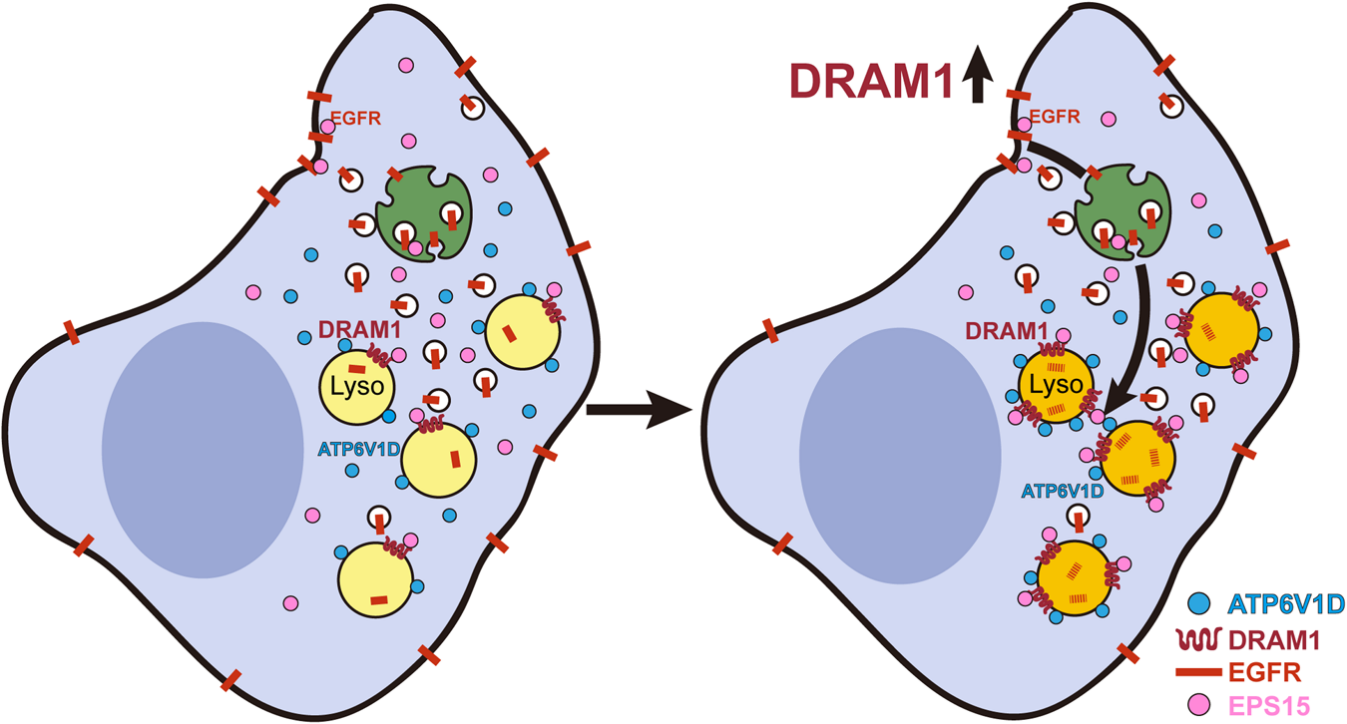
Schematic description of the mechanisms by which DRAM1 regulates EGFR signaling in NSCLC cells. DRAM1 is decreased in NSCLC and overexpression of DRAM1 inhibits tumorigenicity of NSCLC. On one side, DRAM1 interacts with EPS15, therefore accelerates endosomal-lysosomal trafficking of EGFR; on the other, DRAM1 recruits V-ATP6V1D to lysosomes and increases assemble of v-ATPase supercomplex, thus increases lysosomal acidification and lysosomal protease activation. These two actions lead to enhancement of EGFR degradation.

The present work uncovered a novel mechanism of the tumor suppression by DRAM1: promoting EGFR endosomal-lysosomal trafficking and degradation in NSCLC in vitro and in vivo through interacting with EPS15 and promoting the assembly of lysosomal v-ATPase (Fig. 7).

As a known target gene of Tp53, DRAM1 is induced in a Tp53-dependent manner after cellular or genotoxic stress. The ROS–Tp53–DRAM1 pathway is indispensable in mitochondrial dysfunction-triggered autophagy^24,25^. Tp53 mutations occur in many types of cancers, causing them to lose tumor suppressor activity and gain an oncogenic potential. However, no correlation has been observed between DRAM1 expression and Tp53 mutational status, and DRAM1 expression is also partly controlled by the RAS/MAPK pathway in glioblastoma stem cells^26^. Although DRAM1 alone fails to induce programmed cell death under physiological conditions^8,14^, DRAM1-induced autophagy is involved in stimulator-induced apoptosis, where it exerts either a positive or a negative impact on survival of cancer cells. In response to starvation-induced apoptosis and autophagy, DRAM-induced mitophagy or autophagy contributes to apoptosis in hepatocellular carcinoma and breast cancer^27,28^. DRAM1 mediated autophagy can degrade IKKα to aggravate arsenite-induced cytotoxicity^29^. However, DRAM1 is also beneficial to cancer. Highly-expressed DRAM1 is positively associated with shorter overall survival in GBM (glioblastoma multiforme) patients, and knockdown of DRAM1 inhibits p62-mediated autophagy, leading to decreased motility and invasion in GBM stem cells^26^. In addition, DRAM1 promotes the migration and invasion of HepG2 cells via the autophagy-EMT pathway^30^. These contradictory results also imply that the multiple functions of DRAM1 may differ among different types of tumors or different stages of tumorigenesis. Our current results demonstrated that DRAM1 was downregulated in NSCLC patient specimens, and DRAM1 protein was detected in Tp53-wild-type A549 cells, Tp53-null H1299 cells and Tp53-mutant H1975 cells, further indicating that there exists Tp53-independent expression of DRAM1 in NSCLC cells. DRAM1-mediated reduction of mutant EGFR enhanced the cytotoxicity of TKI, and the overexpression of DRAM1 inhibited the growth, migration and invasion of NSCLC in vitro and in vivo, indicating that decrease in DRAM1 expression was beneficial to EGFR mutant NSCLC cells, supporting that DRAM1 has a tumor suppressor activity.

The receptor-mediated endosomal-lysosomal pathway plays a key role in regulating cell surface signaling and the degradation of intracellular components, which are composed of a set of dynamically interconverted intracellular membranous compartments, including early endosomes, recycling endosomes, late endosomes, and lysosomes. EGFR endocytosis was also carried out in the present study to measure the endosomal–lysosomal trafficking route. For wild-type EGFR, the activation and termination of EGFR signaling depend on ligand-stimulated endocytosis and intracellular trafficking. With higher concentrations of ligand (over 20 ng/ml of EGF), EGFR triggers a cascade of responses of downstream signaling pathways, such as RAS/RAF/MEK/ERK, PI3K/Akt/mTOR, and JAK/STAT3^31^, and EGFR itself becomes ubiquitinated, rapidly internalized by the endosomal sorting complex required for transport (ESCRT), then sorted into intraluminal vesicles at multivesicular endosomes (MVEs), and finally degraded in lysosomes following MVE fusion^32^. There are two major destinations for endocytosed EGFR trafficking from early endosomes: recycling to the cell surface or transporting to lysosomes. EGFR mutants impair the interaction with CbI, leading to defective ubiquitination and inefficient lysosomal degradation in NSCLC cells^33^. EGFRvIII, an oncogenic EGFR mutant without exons 2–7 that is universally observed in glioblastoma multiforme, is constitutively active and poorly ubiquitinated, conferring inefficient receptor trafficking to lysosomes and prolonged oncogenic signaling^34^. Reduction of mutant EGFR ubiquitination by CSN6 causes steadily elevated levels of EGFR, leading to proliferation, migration, invasion, and tumorigenesis^35^. In addition to reduced endocytosis of mutant EGFRs, mutant EGFRs are constitutively endocytosed and colocalized with the early/recycling endosomes and the late endosomes in NSCLC cell lines^36^. Mutant EGFRs are preferentially trafficked into the endocytic recycling compartments (ERC), allowing them to go back to the plasma membrane or to interact with Src. Exon-19-deleted EGFR is colocalized with endocytic compartments under steady-state conditions, indicating that the exon-19-deleted EGFR mutant is constantly internalized and sorted to lysosomes for degradation^5^. It has been reported that unliganded EGFR can be internalized at a much slower rate than EGF-stimulated EGFR^37^. Therefore, we used A549 cells with wild-type EGFR and PC9 cells harboring exon-19-deleted EGFR to detect EGFR trafficking and degradation. DRAM1 promoted activated EGFR degradation in PC9 cells and in EGF-stimulated A549 cells, limiting EGFR recycling to the cell surface, resulting in downregulated EGFR levels in EGFR-mutant NSCLC cells and in EGF-stimulated A549 cells.

Protein distribution and structure are vital to predict and analyze a novel function. Compared with the co-IP assay, proximity labeling-based methods coupled with mass spectrometry (MS) offer a higher-throughput approach for systematic analysis of spatially restricted proteomes, especially those proteins localized to discrete subcellular compartments^38^. To find the mechanisms that DRAM1 regulates EGFR trafficking and degradation, BioID2-mediated proximity labeling was performed. BioID2 was fused to the C-terminus of DRAM1 without breaking the signal peptide in the N-terminus. The reported DRAM1-interacted proteins such as SLC1A5, SLC7A5 and SLC3A2^7^ are also included in the list. Intriguingly, we found that DRAM1 interacted with EPS15 (epidermal growth factor receptor pathway substrate 15), a substrate for the tyrosine kinase of EGFR. EPS15 containing the Ub-interacting motif can bind to ubiquitinated EGFR, promoting EGFR internalization and degradation and delaying PI3K-Akt signaling^39^. It has been reported that the prolyl hydroxylase PHD3 acts as a scaffold protein to interact with endocytic adapter EPS15, promotes the internalization of EGFR and decreases EGFR signaling to inhibit cell proliferation and survival of cancer cells^40^. In addition to the trafficking of EGFR, another major process redundantly regulated by EPS15 is the endocytosis of the transferrin receptor. Iron deficiency-induced anemia is present in hematopoietic-specific conditional Eps15/Eps15L1-double-KO mice^41^. The inhibition of EPS15 with an antibody blocks the endocytic pathway and inhibits the internalization of EGF and transferrin^42^. Based on the identified role of EPS15 in vesicle transport, we wondered whether DRAM1 could regulate EGF-mediated endocytosis and the results demonstrated that DRAM1 interacted with EPS15, thus promoted EGFR internalization and trafficking. The significance of EPS15 in DRAM1-mediated trafficking and degradation of EGFR was demonstrated by the EPS15 knockdown study in the present study. The siRNA-induced deficiency in ESP15 blunted DRAM1’s effects on EGFR.

Lysosomes are the main organelles that DRAM1 localized in, and the effect of DRAM1 on lysosomal morphology/position and function was measured. In addition to cargo degradation derived from autophagy, lysosomes are involved in various cell processes, including secretion, plasma membrane repair, signal transduction, energy metabolism, and cell death^43^. DRAM1 potentiates lysosomal destabilization and dell death caused by lysosomal membrane permeabilization (LMP) inducers. In HIV-infected T cells, highly expressed DRAM1 enhances lysosomal membrane permeabilization (LMP) and cathepsin release, leading to mitochondrial outer membrane permeabilization and apoptosis^44^. During 3NP (3-nitropropionic acid)-induced or doxorubicin-induced cell death, DRAM1 recruits BAX to lysosomes to release lysosomal cathepsin B and cleave BID, leading to mitochondria-mediated cell death^13^. The lysosome is a sophisticated signaling center and can be divided into three compartments: highly glycosylated lysosomal membrane proteins, lysosomal proteases in the lumen, and other proteins, such as mTOR, on the lysosomal surface. The present study revealed that as a lysosomal membrane protein, the N-terminus and C-terminus of DRAM1 faced the cytosol, as confirmed in LysoIP assay, TRPML-mediated Ca^2+^ release assay, and acid quenching of GFP.

Research has shown that the peripheral lysosomes and central lysosomes are different in pH. The increased leak permeability to protons and reduced V-ATPase activity lead to reduced acidifying ability of peripheral lysosomes compared with juxtanuclear lysosomes^45^. Furthermore, cells harboring widely distributed lysosomes are characterized by impaired cathepsin maturation and a higher lysosomal pH compared with cells with only peripheral lysosomes^46^. In contrast to control cells, decreased lysosomal pH and increased cathepsin maturation were observed in DRAM1-overexpressing cells. Our previous research found that DRAM1 increased V-ATPase activity^12^, and DRAM1-increased lysosomal acidification was also confirmed in mycobacterial infection^9^. As a heteromultimeric enzyme present in the plasma membrane, endosomes, secretory vesicles and lysosomes, V-ATPase (vacuolar (H^+^)-ATPase) is composed of a peripheral catalytic V1 complex (components A to H) that hydrolyzes ATP and an integral membrane V0 proton pore complex (components a, c, c′, c″, and d) that pumps H^+^ into the lumen of acidic vacuoles or into the extracellular environment; V-ATPase is upregulated in several cancers^47^. To test whether DRAM1 increased lysosomal V-ATPase assembly, lysosomes were isolated, and the V1 and V0 complexes were measured using ATP6V1D and ATP6V0D. Although DRAM1 did not directly interact with the V-ATPase complex, it recruited the V1 subunit and promoted the assembly of V0 and V1 complex, increasing V-ATPase activity to acidify lysosomes. To determine whether DRAM1-induced elevation of lysosomal acidification was responsible for endosomal–lysosomal degradation of EGFR, Baf A1, an inhibitor of V-ATPase, and CQ, a neutralizer of lysosomal acidification, were used. The blockade of EGFR degradation in DRAM1-overexpressing cells was more sensitive to Baf A1 than to CQ.

In summary, this work has identified that DRAM1 is a novel regulator of the endocytosis and lysosomal degradation of EGFR through interacting with EPS15 and promoting the assembly of lysosomal v-ATPase in NSCLC cells. Our data also revealed that DRAM1 may be a potential prognostic biomarker for NSCLC and an indicator for TKI therapy in EGFR-mutant NSCLC. Similarly, other receptors such as PD-L1^48^, androgen receptor^49^, and TLR4 have been reported to be degraded through the endosomal–lysosomal route, thus whether DRAM1 displays specificity for EGFR degradation needs to be further studied.

## Supplementary information

Supplementary material and SI figure legends

Supplementary table 1

Supplementary table 2

Sipplementary table 3

Figure SI 1

Figure SI 2

Figure SI 3

Figure SI 4

Figure SI 5

Figure SI 6

Figure SI 7

Figure SI 8

Figure SI 9

Figure SI 10
