# Supplementary material for: DRAM1 plays a tumor suppressor role in NSCLC cells by promoting lysosomal degradation of EGFR
Source: Cell Death Dis. 2020 Sep 17;11(9):768. doi: 10.1038/s41419-020-02979-9 (PMC7498585; doi:10.1038/s41419-020-02979-9)
Supplement: Supplementary file 1 — Supplementary material and SI figure legends [file 41419_2020_2979_MOESM1_ESM.docx]

**Supplementary material**

**Materials and Methods**

***Knockdown of DRAM1***

MCF-7 cells and Hela cells were cultured in DMEM (Sigma) with 10% fatal bovine serum (SA015, SERANA). The sequences of DRAM1 siRNA are as follows: control siRNA (UUCUCUCCGAACGUGUCACGUTT), siDRAM1-1 (AGCCACGAUGUACAAGATT), siDRAM1-2 (CCACAGAAAUCAAUGGUGATT). Cells with appropriate density were cultured in 24-well plates on the day prior to RNAi experiment. Lipofectamine RNAiMAX reagent (0.5 μl) was diluted with 25 μl Opti-MEM, and gently mixed with 25 μl Opti-MEM medium containing 40 nM siRNA. Then, the mixture was placed at room temperature for 5 min to form a siRNA/lipofectamine RNAiMAX complex. Cells were replaced with fresh medium and added with the above-mentioned mixture for further 72 h before experiments.

***Cell proliferation assay***

PC9 cells and DRAM1-overexpressing PC9 cells were seeded at a density of 3000 cells per well. After 24 h, cells were treated with 2 nM Baf A1 and 100 nM CQ for another 48 h, 10 μl of CCK-8 was added into each well to measure cellular viability. PC9 and DRAM1-overexpressing PC9 cells were transfected si-EPS15 using RNAiMax (Lipofectamine™ RNAiMAX Transfection Reagent, Thermofisher) for 24 h followed by plating into 96-well plates and growth for 72 h, cell viability was measured as above-mentioned.

***Colony formation assay***

H1975 control cells, DRAM1-overexpressing H1975 cells, PC9 control cells, DRAM1-overexpressing PC9 cells, PC9-negative cells and DRAM1-knockdown PC9 cells were seeded into 6-well plates at a density of 200 cells per well and colonies were counted after 2 weeks.

***Wound healing assay***

Wound healing assays are widely conducted to study cell migration and cell-cell interactions. Cells were seeded at 3×10^5^ cells/well in a 6-well plate in 2 ml of RPMI-1640 medium containing 10% FBS. After 24 h, the cells reached 80% confluence as a monolayer. A new 20 μl pipette tip was gently and slowly scratched on the monolayer across the center of the well. The well was gently washed twice with medium to remove the detached cells. Cells were cultured in fresh serum-free medium or containing 2 nM Baf A1 for an additional 48 h. Cells were washed twice with 1× PBS and fixed for 10 min with 4% paraformaldehyde.

***Transwell assay***

Transwell assays are often used to detect cell migration and invasion. For migration, 600 μl RPMI-1640 medium containing 10% FBS or 2 nM Baf A1 contained RPMI-1640 were added to the lower compartment of a Transwell plate (#3422, Costar) with an 8 μm pore insert, and 8×10^4^ cells in RPMI-1640 or 2 nM Baf A1 contained RPMI-1640 were added to the upper compartment and incubated at 37°C for 12 h. For invasion, Matrigel was diluted to 1 mg/ml in serum free-cold RPMI-1640, and 100 μl of this dilutant was added into the insert to incubate 4 h for gelling. Then, 8×10^4^ cells in RPMI-1640 or 2 nM Baf A1 contained RPMI-1640 were added to the upper compartment and incubated at 37°C for 24 h. Cells in the lower chamber were fixed with 4% paraformaldehyde at room temperature for 10 min and stained with 1% crystal violet for 20 min. The cells in the lower chamber were photographed under a light microscope.

***LysoTracker Green staining***

LysoTracker Green DND-26 (40738ES50, Yeason, China) is a green fluorescent dye that stains acidic compartments in live cells and is used to detect the distribution of lysosomes. Cells were stained with 50 nM LysoTracker Green diluted in RPMI-1640 at room temperature for 30 min, washed twice with PBS, and cultured with RPMI-1640. Images were taken using a confocal microscope (Zeiss, Germany).

***Fluorescence-based EGFR trafficking assays***

DRAM1-knockdown cells were cultured in serum-free medium overnight and stimulated with 100 ng/ml Texas red conjugated EGF for 30 min at 4°C, then washed and incubated at 37°C for additional time. To visualize the colocalization between EGFR and early endosomes, cells underwent above experiment were fixed and immunostained with anti-EEA1, a marker of early endosome. To visualize the colocalization between EGFR and lysosomes, cells underwent above experiment were stained with 50 nM Lysotracker Green for 30 min on ice. Fluorescence pictures were captured by LSM-710 confocal microscope (Zeiss, Germany).

**Supplementary Figure**

**Figure SI. 1 Morphology changes in DRAM1-deficient cells.**

(A) Fluorescent images showing GFP from lenti-GFP vector cells and lenti-DRAM1 cells after 72 h post-transfection. (B) Fluorescent images and phase contrast microscopy images showing mCherry from lenti-mCherry vector cells and lenti-DRAM1 cells. (C) Contrast microscopy images and fluorescence microscopy images showing mCherry from lenti-sh vector cells and lenti-shDRAM1 cells. The efficiency of knockdown was measured using RT-qPCR. ****P* < 0.005.

**Figure SI. 2 DRAM1 inhibits NSCLC cell growth, migration and invasion.**

(A) Representative images of colony formation assays of DRAM1-overexpressing and DRAM1-knockdown NSCLC cells. The number of colony was counted. (B) Representative images of the wound healing assay of the indicated cells. (C-D) Migration and invasion ability of indicated cells measured using a Transwell migration assay and Matrigel invasion assay. The number of migrated or invaded cells was counted per picture. (E) Western blot analysis of E-cadherin, α-SMA and N-cadherin in DRAM1-overexpressing and DRAM1-knockdown NSCLC cells. Data are shown as the mean ± SD of three independent experiments. **P* < 0.05, ***P* < 0.01.

**Figure SI. 3 DRAM1 decreases EGFR protein levels without increasing EGFR secretion.**

(A) The blot quantification of Figure 3A was performed by densitometry. (B) Western blots in Figure 3C were quantified by densitometry (n = 4). (C) Immunohistochemical staining of DRAM1 and EGFR in lung cancer tissues derived from 12 patients. Scale bar is 200 μm. (D) Western blot analysis of CD63, ALIX, TSG101 and SDCBP in H1975 cells, H1975-DRAM1 cells, PC9 cells and PC9-DRAM1 cells. (E) Cells were pretreated with DRAM1 siRNA and control siRNA. After 48 h, cells were starved overnight and stimulated with 100 ng/ml EGF for indicated times to assess EGFR levels. EGFR protein was detected using Western blotting. The quantification was performed by densitometry. **P* < 0.05, ***P* < 0.01.

**Figure SI. 4 Knockdown of DRAM1 impairs EGFR endocytic trafficking.**

(A) The subcellular distribution of Texas Red-EGF and colocalization with EEA1, a marker of early endosomes, and lysosomes in DRAM1-knockdown cells and control cells were revealed with a confocal microscopy. Arrows indicated merged foci. Scale bar: 5 μm. (B) Cells were pretreated with DRAM1 siRNA and control siRNA. After 48 h, cells were serum-starved overnight and stimulated with 100 ng/ml Texas Red-EGF for 30 min, cells were then washed and cultured for indicated time before being fixed and analyzed using microscopy. Scale bar: 10 μm. Data were expressed as the mean ± SD. **P* < 0.05.

**Figure SI. 5 The colocalization between EGFR and RAB5 and RAB7 in DRAM1 overexpressing A549 cells.**

(A-B) A549 cells and A549-DRAM1 cells were serum-starved overnight and treated with 100 ng/ml Alexa Fluor 488-EGF at 4°C for 30 min, washed and incubated for 10 min, 20 min and 30 min before being fixed and coimmunostained with RAB5 (gray) or RAB7 (gray). Scale bar: 10 μm.

**Figure SI. 6 DRAM1 mainly locates in lysosomes and N- and C-terminal of DRAM1 orients to the cytosol.**

(A-H) A549 cells were transiently cotransfected with 3FLAG-DRAM1 plasmid and mCherry-LAMP1, mTurquoise2-Golgi, mCherry-ER, mTurquoise2-Mitochondria, mCherry-RAB5, GFP-RAB7, GFP-RAB9, or DsRed-RAB11. The intensity of immunofluorescence was analyzed. Scale bar: 10 μm. (I) HEK293T cells were cotransfected with 3FLAG-DRAM1 plasmid and TMEM192-3HA plasmid. Anti-FLAG agarose and anti-HA-magnetic beads were applied to isolate lysosomes. The proteins of LAMP1, calnexin, HA, FLAG and tubulin were measured using Western blot analysis. (J-K) A549 cells were transfected with 3FLAG-DRAM1-GCaMP6 plasmid for 24 h, fluorescence pictures were taken with fluorescence microscope, and fluorescence intensity was measured using a fluorescence microplate reader (excitation 488 nm/emission 550 nm) before and after adding 20 μM of ML-SA1 for 10 min. Scale bar: 25 μm. (L) A549 cells were transfected with DRAM1-GFP plasmid for 24 h followed by LysoTracker Red staining to indicate lysosomes. Fluorescence pictures were taken using fluorescence microscopy. Scale bar: 10 μm. Data were shown as the means ± SD of three independent experiments. ***P* < 0.01.

**Figure SI. 7 All identified proteins in BioID2 assay.**

(A) All identified proteins by LC-MS. (B-C) Functional annotation of identified biotinylated proteins (log2 > 2) based on KEGG pathways and biological processes (BP) were analyzed by using the DAVID database.

**Figure SI. 8 Knockdown of DRAM1 diminishes the mature of cathepsin D.**

(A) LysoTracker Green was quantified as the mean fluorescence intensity (MFI)/10^4^ cells using a microplate reader. (B) DRAM1 was knocked down in MCF-7 and Hela cells using siRNA, cathepsin D was detected using Western blot analysis.

**Figure SI. 9 DRAM1 promotes the translocation of** **V-ATP6V1D to lysosomes in NSCLC cells.**

(A) Co-IP assay was performed with lysates of H1975 cells overexpressing DRAM1 to detect the direct interaction between DRAM1 and V-ATP6V0D or V-ATP6V1D. The levels of endogenous V-ATP6V0D and V-ATP6V1D were detected by Western blot analysis. (B-C) Immunofluorescence of H1975 cells cotransfected with 3FLAG-DRAM1 (green) and mCherry-LAMP1 (red) and coimmunostained with V-ATP6V0D (gray) or V-ATP6V1D (gray). (D-E) Immunofluorescence of PC9 cells cotransfected with 3FLAG-DRAM1 (green) and mCherry-LAMP1 (red) and coimmunostained with V-ATP6V0D (gray) or V-ATP6V1D (gray). Scale bar: 10 μm.

**Figure SI. 10 Inhibition of proliferation, migration and invasion by DRAM1 depends on lysosomal acidification and EPS15 in NSCLC.**

PC9 cells and DRAM1-overexpressing PC9 cells were treated with 2 nM Baf A1, 100 nM CQ or si-EPS15 for 48 h. (A) Cell viability was measured using CCK-8. (B) After incubating 2 nM Baf A1 for 12 h or decreasing EPS15, migration was detected using Transwell assay. (C) After incubating 2 nM Baf A1 for 24 h or decreasing EPS15 using siRNA, invasion was detected using Transwell assay. (D) cell migration was measured using wound healing assay. Scale bar: 200 μm. Data were shown as the means ± SD. **P* < 0.05 compared to DRAM1-overexpressing PC9 cells.
