# Supplementary material for: DRAM1 plays a tumor suppressor role in NSCLC cells by promoting lysosomal degradation of EGFR
Source: Cell Death Dis. 2020 Sep 17;11(9):768. doi: 10.1038/s41419-020-02979-9 (PMC7498585; doi:10.1038/s41419-020-02979-9)
Supplement: Supplementary file 2 — Supplementary table 1 [file 41419_2020_2979_MOESM2_ESM.docx]

**Supplementary Table 1. The information of plasmids used to label organelles**

| Plasmid name | Product code |
| --- | --- |
| pmTurquoise2-Golgi | Addgene (36205) |
| mcherry-ER | Addgene (55041) |
| pmTurquoise2-Mito | Addgene (36208) |
| mcherry-RAB5 | Addgene (49201) |
| GFP-RAB7 | Addgene (12605) |
| GFP-RAB9 | Addgene (12663) |
| DsRed-RAB11 | Addgene (12679) |
