# Supplementary material for: DRAM1 plays a tumor suppressor role in NSCLC cells by promoting lysosomal degradation of EGFR
Source: Cell Death Dis. 2020 Sep 17;11(9):768. doi: 10.1038/s41419-020-02979-9 (PMC7498585; doi:10.1038/s41419-020-02979-9)
Supplement: Supplementary file 3 — Supplementary table 2 [file 41419_2020_2979_MOESM3_ESM.docx]

**Supplementary Table 2. List of antibodies used in Western blotting**

| **Antibody name** | **Company** | **Dilution** |
| --- | --- | --- |
| Anti-DRAM1 | Abcam (ab88648) | 1:1000 |
| Anti-RAB7 | Abcam (ab126712) | 1:1000 |
| Anti-RAB9 | Abcam (ab179815) | 1:1000 |
| Anti-ALIX | Abcam (ab186429) | 1:1000 |
| Anti-TSG101 | Abcam (ab125011) | 1:1000 |
| Anti-V-ATP6V0D | Abcam (ab202899) | 1:1000 |
| Anti-V-ATP6V1D | Abcam (ab157458) | 1:1000 |
| Anti-EPS15 | Abcam (ab174291) | 1:1000 |
| Anti-α-tubulin | Proteintech (66031-1-Ig) | 1:4000 |
| Anti-β-actin | Proteintech (60008-1-Ig) | 1:5000 |
| Anti-CD63 | Proteintech (25682-1-AP) | 1:500 |
| Anti-SDCBP | Proteintech (22399-1-AP) | 1:500 |
| Anti-EGFR | Santa Cruz Biotechnology (sc-03) | 1:1000 |
| Anti-Cathepsin B | Santa Cruz Biotechnology (sc-6490) | 1:500 |
| Anti-Cathepsin D | Santa Cruz Biotechnology (sc-10725) | 1:500 |
| Anti-ERK1/2 | Santa Cruz Biotechnology (sc-514302) | 1:1000 |
| Anti-PCNA | Santa Cruz Biotechnology (sc-7907) | 1:100 |
| Anti-p-EGFR (Tyr1068) | Cell Signaling Technology (#3777) | 1:1000 |
| Anti-E-Cadherin | Cell Signaling Technology (#3195) | 1:1000 |
| Anti-α-SMA | Cell Signaling Technology (#56856) | 1:1000 |
| Anti-N-Cadherin | Cell Signaling Technology (#4061) | 1:1000 |
| Anti-Biotin | Cell Signaling Technology (#5597) | 1:1000 |
| Anti-AKT | Cell Signaling Technology (#4691) | 1:1000 |
| Anti-p-AKT (Ser473) | Cell Signaling Technology (#4060) | 1:1000 |
| Anti-p-ERK1/2 (Thr202/Tyr204) | Cell Signaling Technology (#4370) | 1:1000 |
| Anti-p-STAT3 (Tyr705) | Cell Signaling Technology (#9145) | 1:1000 |
| Anti-Calnexin | Cell Signaling Technology (#2679) | 1:1000 |
| Anti-HA | Cell Signaling Technology (#3724) | 1:1000 |
| Anti-FLAG | Sigma-Aldrich (F3165) | 1:1000 |
