# Supplementary material for: DRAM1 plays a tumor suppressor role in NSCLC cells by promoting lysosomal degradation of EGFR
Source: Cell Death Dis. 2020 Sep 17;11(9):768. doi: 10.1038/s41419-020-02979-9 (PMC7498585; doi:10.1038/s41419-020-02979-9)
Supplement: Supplementary file 4 — Sipplementary table 3 [file 41419_2020_2979_MOESM4_ESM.docx]

**Supplementary Table 3. List of identified proteins**

| Accession | Description | Gene name | # PSM C-1 | # PSM D-1 | # PSM C-2 | # PSM D-2 | # AAs | MW [kDa] | calc. pI |
| --- | --- | --- | --- | --- | --- | --- | --- | --- | --- |
| O95490-7 | Isoform 7 of Adhesion G protein-coupled receptor L2 | ADGRL2 | 5 | 24 | 3 | 13 | 1461 | 163.3 | 6.14 |
| P55011 | Solute carrier family 12 member 2 | SLC12A2 | 6 | 48 | 3 | 30 | 1212 | 131.4 | 6.40 |
| P17301 | Integrin alpha-2 | ITGA2 | 1 | 36 | 2 | 24 | 1181 | 129.2 | 5.31 |
| Q9Y6M7-6 | Isoform 6 of Sodium bicarbonate cotransporter 3 | SLC4A7 | 5 | 43 | 5 | 50 | 1131 | 127.3 | 6.76 |
| Q9BSJ8-2 | Isoform 2 of Extended synaptotagmin-1 | ESYT1 | 4 | 92 | 4 | 30 | 1114 | 123.9 | 5.78 |
| Q14126 | Desmoglein-2 | DSG2 | 6 | 33 | 1 | 24 | 1118 | 122.2 | 5.24 |
| P23229-5 | Isoform Alpha-6X2B of Integrin alpha-6 | ITGA6 | 0 | 31 | 5 | 31 | 1086 | 121.6 | 7.36 |
| Q5T5C0-3 | Isoform 3 of Syntaxin-binding protein 5 | STXBP5 | 0 | 26 | 1 | 5 | 1098 | 121.5 | 6.96 |
| Q9UIQ6 | Leucyl-cystinyl aminopeptidase | LNPEP | 4 | 54 | 3 | 30 | 1025 | 117.3 | 5.73 |
| P06756 | Integrin alpha-V | ITGAV | 1 | 20 | 4 | 25 | 1048 | 116.0 | 5.68 |
| Q8IZA0 | Dyslexia-associated protein KIAA0319-like protein | KIAA0319L | 2 | 16 | 5 | 20 | 1049 | 115.6 | 6.05 |
| P13612 | Integrin alpha-4 | ITGA4 | 0 | 27 | 2 | 26 | 1032 | 114.8 | 6.48 |
| P08648 | Integrin alpha-5 | ITGA5 | 0 | 23 | 5 | 34 | 1049 | 114.5 | 5.77 |
| Q8N766 | ER membrane protein complex subunit 1 | EMC1 | 5 | 51 | 8 | 75 | 993 | 111.7 | 7.66 |
| P42566 | Epidermal growth factor receptor substrate 15 | EPS15 | 1 | 16 | 1 | 20 | 896 | 98.6 | 4.64 |
| Q14118 | Dystroglycan | DAG1 | 3 | 25 | 1 | 29 | 895 | 97.4 | 8.56 |
| Q8IWT6 | Volume-regulated anion channel subunit LRRC8A | LRRC8A | 1 | 14 | 1 | 32 | 810 | 94.1 | 7.94 |
| Q8TD16 | Protein bicaudal D homolog 2 | BICD2 | 4 | 38 | 0 | 6 | 824 | 93.5 | 5.44 |
| Q8TDW0 | Volume-regulated anion channel subunit LRRC8C | LRRC8C | 1 | 15 | 1 | 28 | 803 | 92.4 | 7.62 |
| P05556 | Integrin beta-1 | ITGB1 | 3 | 62 | 7 | 62 | 798 | 88.4 | 5.39 |
| P18084 | Integrin beta-5 | ITGB5 | 2 | 12 | 2 | 12 | 799 | 88.0 | 6.06 |
| P42892 | Endothelin-converting enzyme 1 | ECE1 | 2 | 25 | 3 | 40 | 770 | 87.1 | 5.88 |
| P05067 | Amyloid-beta A4 protein | APP | 3 | 20 | 4 | 20 | 770 | 86.9 | 4.82 |
| Q96PD2 | Discoidin, CUB and LCCL domain-containing protein 2 | DCBLD2 | 0 | 11 | 2 | 8 | 775 | 85.0 | 7.17 |
| P13807 | Glycogen [starch] synthase | GYS1 | 6 | 24 | 1 | 5 | 737 | 83.7 | 6.18 |
| Q8TBA6 | Golgin subfamily A member 5 | GOLGA5 | 4 | 35 | 3 | 13 | 731 | 83.0 | 5.83 |
| Q9H2J7 | Sodium-dependent neutral amino acid transporter B(0)AT2 | SLC6A15 | 1 | 14 | 1 | 21 | 730 | 81.8 | 5.19 |
| Q13586 | Stromal interaction molecule 1 | STIM1 | 2 | 58 | 4 | 45 | 685 | 77.4 | 6.67 |
| O15228 | Dihydroxyacetone phosphate acyltransferase | GNPAT | 0 | 4 | 2 | 8 | 680 | 77.1 | 6.57 |
| Q99805 | Transmembrane 9 superfamily member 2 | TM9SF2 | 2 | 11 | 1 | 4 | 663 | 75.7 | 7.44 |
| O96005-4 | Isoform 3 of Cleft lip and palate transmembrane protein 1 | CLPTM1 | 0 | 9 | 1 | 18 | 655 | 75.1 | 6.64 |
| Q92544 | Transmembrane 9 superfamily member 4 | TM9SF4 | 2 | 8 | 1 | 4 | 642 | 74.5 | 6.54 |
| Q9H267 | Vacuolar protein sorting-associated protein 33B | VPS33B | 2 | 22 | 1 | 5 | 617 | 70.5 | 6.71 |
| Q9UJ14 | Glutathione hydrolase 7 | GGT7 | 2 | 17 | 2 | 18 | 662 | 70.4 | 5.10 |
| O14967 | Calmegin | CLGN | 2 | 31 | 3 | 12 | 610 | 70.0 | 4.69 |
| P08240 | Signal recognition particle receptor subunit alpha | SRPRA | 5 | 21 | 2 | 20 | 638 | 69.8 | 8.95 |
| P08195 | 4F2 cell-surface antigen heavy chain | SLC3A2 | 14 | 66 | 10 | 56 | 630 | 68.0 | 5.01 |
| Q9HD45 | Transmembrane 9 superfamily member 3 | TM9SF3 | 1 | 13 | 3 | 13 | 589 | 67.8 | 7.21 |
| A1L0T0 | Acetolactate synthase-like protein | ILVBL | 0 | 7 | 2 | 8 | 632 | 67.8 | 8.15 |
| Q9NTJ5 | Phosphatidylinositide phosphatase SAC1 | SACM1L | 0 | 6 | 3 | 17 | 587 | 66.9 | 7.12 |
| Q5JTV8-3 | Isoform 3 of Torsin-1A-interacting protein 1 | TOR1AIP1 | 4 | 23 | 4 | 20 | 584 | 66.3 | 8.18 |
| Q9BTX1-6 | Isoform 6 of Nucleoporin NDC1 | NDC1 | 0 | 8 | 2 | 8 | 559 | 63.1 | 9.07 |
| Q8TBZ3 | WD repeat-containing protein 20 | WDR20 | 2 | 12 | 2 | 8 | 569 | 62.9 | 8.00 |
| Q96S66 | Chloride channel CLIC-like protein 1 | CLCC1 | 2 | 32 | 3 | 52 | 551 | 62.0 | 5.55 |
| O95817 | BAG family molecular chaperone regulator 3 | BAG3 | 0 | 6 | 2 | 8 | 575 | 61.6 | 6.95 |
| Q9NQS3 | Nectin-3 | NECTIN3 | 0 | 13 | 3 | 14 | 549 | 61.0 | 6.19 |
| P49591 | Serine--tRNA ligase | SARS | 1 | 4 | 0 | 4 | 514 | 58.7 | 6.43 |
| Q92692 | Nectin-2 | NECTIN2 | 0 | 15 | 0 | 17 | 538 | 57.7 | 4.82 |
| Q15223 | Nectin-1 | NECTIN1 | 1 | 6 | 1 | 14 | 517 | 57.1 | 6.10 |
| Q92575 | UBX domain-containing protein 4 | UBXN4 | 1 | 29 | 4 | 17 | 508 | 56.7 | 6.38 |
| Q15758 | Neutral amino acid transporter B(0) | SLC1A5 | 10 | 43 | 5 | 23 | 541 | 56.6 | 5.48 |
| Q7Z434 | Mitochondrial antiviral-signaling protein | MAVS | 0 | 8 | 1 | 5 | 540 | 56.5 | 5.52 |
| Q96QD8 | Sodium-coupled neutral amino acid transporter 2 | SLC38A2 | 1 | 12 | 2 | 13 | 506 | 56.0 | 8.00 |
| Q96A33 | Coiled-coil domain-containing protein 47 | CCDC47 | 2 | 77 | 5 | 94 | 483 | 55.8 | 4.87 |
| Q99624 | Sodium-coupled neutral amino acid transporter 3 | SLC38A3 | 1 | 7 | 1 | 4 | 504 | 55.7 | 7.91 |
| Q9NRY5 | Protein FAM114A2 | FAM114A2 | 2 | 21 | 1 | 8 | 505 | 55.4 | 4.88 |
| Q9UPY5 | Cystine/glutamate transporter | SLC7A11 | 0 | 6 | 1 | 9 | 501 | 55.4 | 9.19 |
| Q01650 | Large neutral amino acids transporter small subunit 1 | SLC7A5 | 1 | 19 | 1 | 24 | 507 | 55.0 | 7.72 |
| O75844 | CAAX prenyl protease 1 homolog | ZMPSTE24 | 2 | 8 | 0 | 9 | 475 | 54.8 | 7.49 |
| Q8IV08 | Phospholipase D3 | PLD3 | 1 | 5 | 0 | 12 | 490 | 54.7 | 6.47 |
| Q658P3-3 | Isoform 3 of Metalloreductase STEAP3 | STEAP3 | 0 | 12 | 0 | 7 | 487 | 54.4 | 8.72 |
| P11166 | Solute carrier family 2, facilitated glucose transporter member 1 | SLC2A1 | 1 | 5 | 1 | 9 | 492 | 54.0 | 8.72 |
| Q9H2H9 | Sodium-coupled neutral amino acid transporter 1 | SLC38A1 | 2 | 11 | 4 | 18 | 487 | 54.0 | 7.02 |
| P53985 | Monocarboxylate transporter 1 | SLC16A1 | 2 | 18 | 1 | 17 | 500 | 53.9 | 8.66 |
| Q8WTV0-4 | Isoform 4 of Scavenger receptor class B member 1 | SCARB1 | 0 | 21 | 1 | 13 | 474 | 53.5 | 7.25 |
| Q9UBB4 | Ataxin-10 | ATXN10 | 0 | 5 | 1 | 4 | 475 | 53.5 | 5.25 |
| Q5ZPR3-3 | Isoform 3 of CD276 antigen | CD276 | 0 | 4 | 0 | 5 | 493 | 52.7 | 5.52 |
| P60228 | Eukaryotic translation initiation factor 3 subunit E | EIF3E | 3 | 19 | 1 | 4 | 445 | 52.2 | 6.04 |
| Q96JJ7 | Protein disulfide-isomerase TMX3 | TMX3 | 0 | 16 | 1 | 12 | 454 | 51.8 | 4.91 |
| Q92692-2 | Isoform Alpha of Nectin-2 | NECTIN2 | 1 | 12 | 1 | 18 | 479 | 51.3 | 5.11 |
| Q8NFQ8 | Torsin-1A-interacting protein 2 | TOR1AIP2 | 2 | 25 | 2 | 13 | 470 | 51.2 | 4.96 |
| P42167 | Lamina-associated polypeptide 2, isoforms beta/gamma | TMPO | 2 | 19 | 7 | 39 | 454 | 50.6 | 9.38 |
| P49821-2 | Isoform 2 of NADH dehydrogenase [ubiquinone] flavoprotein 1 | NDUFV1 | 1 | 9 | 1 | 6 | 455 | 49.8 | 8.21 |
| Q13501 | Sequestosome-1 | SQSTM1 | 1 | 8 | 0 | 4 | 440 | 47.7 | 5.22 |
| Q9Y6K0 | Choline/ethanolaminephosphotransferase 1 | CEPT1 | 0 | 4 | 0 | 4 | 416 | 46.5 | 8.21 |
| P49768-6 | Isoform 6 of Presenilin-1 | PSEN1 | 1 | 6 | 1 | 7 | 409 | 46.3 | 6.73 |
| Q99442 | Translocation protein SEC62 | SEC62 | 2 | 11 | 0 | 6 | 399 | 45.8 | 7.12 |
| Q9HCU5 | Prolactin regulatory element-binding protein | PREB | 0 | 12 | 1 | 11 | 417 | 45.4 | 7.88 |
| Q96G23 | Ceramide synthase 2 | CERS2 | 1 | 11 | 1 | 9 | 380 | 44.8 | 8.98 |
| Q5JVF3-3 | Isoform 3 of PCI domain-containing protein 2 | PCID2 | 3 | 24 | 3 | 20 | 376 | 43.3 | 8.63 |
| P37268-5 | Isoform 5 of Squalene synthase | FDFT1 | 1 | 4 | 1 | 4 | 374 | 43.0 | 6.79 |
| O43837 | Isocitrate dehydrogenase [NAD] subunit beta | IDH3B | 2 | 24 | 1 | 21 | 385 | 42.2 | 8.46 |
| O43869 | Olfactory receptor 2T1 | OR2T1 | 4 | 39 | 1 | 51 | 369 | 42.0 | 8.38 |
| Q15738 | Sterol-4-alpha-carboxylate 3-dehydrogenase | NSDHL | 2 | 9 | 1 | 7 | 373 | 41.9 | 8.06 |
| Q8NEW0 | Zinc transporter 7 | SLC30A7 | 0 | 11 | 0 | 9 | 376 | 41.6 | 6.95 |
| Q9Y679-3 | Isoform 3 of Ancient ubiquitous protein 1 | AUP1 | 0 | 7 | 1 | 8 | 373 | 41.4 | 7.90 |
| P01892 | HLA class I histocompatibility antigen, A-2 alpha chain | HLA-A | 0 | 17 | 1 | 19 | 365 | 40.9 | 6.99 |
| Q8NBU5 | ATPase family AAA domain-containing protein 1 | ATAD1 | 1 | 6 | 0 | 5 | 361 | 40.7 | 6.90 |
| Q9NV96 | Cell cycle control protein 50A | TMEM30A | 0 | 6 | 2 | 8 | 361 | 40.7 | 8.59 |
| P78310 | Coxsackievirus and adenovirus receptor | CXADR | 1 | 44 | 1 | 49 | 365 | 40.0 | 7.56 |
| O95210 | Starch-binding domain-containing protein 1 | STBD1 | 2 | 20 | 1 | 4 | 358 | 39.0 | 5.73 |
| P48729 | Casein kinase I isoform alpha | CSNK1A1 | 1 | 4 | 1 | 6 | 337 | 38.9 | 9.57 |
| B7ZAQ6-2 | Isoform 2 of Golgi pH regulator A | GPR89A | 1 | 7 | 1 | 8 | 335 | 38.5 | 9.35 |
| Q9Y394 | Dehydrogenase/reductase SDR family member 7 | DHRS7 | 2 | 16 | 1 | 10 | 339 | 38.3 | 8.32 |
| O14828 | Secretory carrier-associated membrane protein 3 | SCAMP3 | 0 | 10 | 0 | 10 | 347 | 38.3 | 7.64 |
| P98172 | Ephrin-B1 | EFNB1 | 1 | 25 | 3 | 17 | 346 | 38.0 | 8.94 |
| O15126 | Secretory carrier-associated membrane protein 1 | SCAMP1 | 0 | 9 | 0 | 12 | 338 | 37.9 | 7.42 |
| Q9P289-2 | Isoform 2 of Serine/threonine-protein kinase 26 | STK26 | 1 | 9 | 1 | 5 | 339 | 37.7 | 6.09 |
| P62136 | Serine/threonine-protein phosphatase PP1-alpha catalytic subunit | PPP1CA | 4 | 22 | 0 | 4 | 330 | 37.5 | 6.33 |
| Q9BQE5 | Apolipoprotein L2 | APOL2 | 0 | 4 | 0 | 5 | 337 | 37.1 | 6.74 |
| Q14257 | Reticulocalbin-2 | RCN2 | 0 | 8 | 0 | 6 | 317 | 36.9 | 4.40 |
| Q9Y385 | Ubiquitin-conjugating enzyme E2 J1 | UBE2J1 | 1 | 7 | 1 | 10 | 318 | 35.2 | 6.74 |
| Q9HC07 | Transmembrane protein 165 | TMEM165 | 1 | 4 | 0 | 6 | 324 | 34.9 | 7.02 |
| Q15006 | ER membrane protein complex subunit 2 | EMC2 | 0 | 21 | 2 | 19 | 297 | 34.8 | 6.57 |
| Q9Y6I9 | Testis-expressed protein 264 | TEX264 | 0 | 12 | 1 | 9 | 313 | 34.2 | 4.86 |
| P53007 | Tricarboxylate transport protein | SLC25A1 | 1 | 8 | 1 | 11 | 311 | 34.0 | 9.89 |
| Q8TC12-2 | Isoform 2 of Retinol dehydrogenase 11 | RDH11 | 0 | 7 | 1 | 4 | 305 | 34.0 | 8.69 |
| Q9Y6C9 | Mitochondrial carrier homolog 2 | MTCH2 | 2 | 10 | 1 | 13 | 303 | 33.3 | 7.97 |
| P49069 | Calcium signal-modulating cyclophilin ligand | CAMLG | 0 | 6 | 2 | 8 | 296 | 32.9 | 8.05 |
| Q14165 | Malectin | MLEC | 0 | 6 | 2 | 11 | 292 | 32.2 | 5.41 |
| Q9H3N1 | Thioredoxin-related transmembrane protein 1 | TMX1 | 3 | 43 | 3 | 41 | 280 | 31.8 | 4.98 |
| Q16625-4 | Isoform 4 of Occludin | OCLN | 2 | 8 | 1 | 7 | 271 | 31.6 | 5.68 |
| Q9NUM4 | Transmembrane protein 106B | TMEM106B | 0 | 4 | 0 | 7 | 274 | 31.1 | 6.99 |
| Q9NQX7 | Integral membrane protein 2C | ITM2C | 2 | 9 | 2 | 12 | 267 | 30.2 | 8.00 |
| Q9P0I2 | ER membrane protein complex subunit 3 | EMC3 | 1 | 8 | 1 | 21 | 261 | 29.9 | 6.81 |
| Q9Y5M8 | Signal recognition particle receptor subunit beta | SRPRB | 2 | 18 | 2 | 16 | 271 | 29.7 | 9.04 |
| P25789 | Proteasome subunit alpha type-4 | PSMA4 | 1 | 4 | 1 | 5 | 261 | 29.5 | 7.72 |
| O95297-2 | Isoform 2 of Myelin protein zero-like protein 1 | MPZL1 | 2 | 14 | 2 | 14 | 268 | 29.0 | 8.72 |
| O75431-2 | Isoform 2 of Metaxin-2 | MTX2 | 1 | 7 | 1 | 5 | 253 | 28.8 | 6.54 |
| Q9H8H3 | Methyltransferase-like protein 7A | METTL7A | 0 | 4 | 0 | 4 | 244 | 28.3 | 8.38 |
| Q9P0L0 | Vesicle-associated membrane protein-associated protein A | VAPA | 0 | 18 | 1 | 18 | 249 | 27.9 | 8.62 |
| O95292 | Vesicle-associated membrane protein-associated protein B/C | VAPB | 1 | 24 | 1 | 19 | 243 | 27.2 | 7.30 |
| Q5UCC4-2 | Isoform 2 of ER membrane protein complex subunit 10 | EMC10 | 1 | 5 | 0 | 5 | 254 | 26.8 | 6.29 |
| Q9UEU0 | Vesicle transport through interaction with t-SNAREs homolog 1B | VTI1B | 1 | 5 | 0 | 5 | 232 | 26.7 | 9.04 |
| Q9NPA0 | ER membrane protein complex subunit 7 | EMC7 | 0 | 16 | 2 | 20 | 242 | 26.5 | 9.25 |
| Q8N682 | DNA damage-regulated autophagy modulator protein 1 | DRAM1 | 0 | 39 | 1 | 31 | 238 | 26.2 | 6.84 |
| Q9BVC6 | Transmembrane protein 109 | TMEM109 | 1 | 4 | 0 | 4 | 243 | 26.2 | 10.48 |
| Q9Y3A6 | Transmembrane emp24 domain-containing protein 5 | TMED5 | 1 | 5 | 1 | 4 | 229 | 26.0 | 4.84 |
| P00403 | Cytochrome c oxidase subunit 2 | MT-CO2 | 2 | 11 | 1 | 5 | 227 | 25.5 | 4.82 |
| Q9Y2Q3 | Glutathione S-transferase kappa 1 | GSTK1 | 0 | 13 | 1 | 6 | 226 | 25.5 | 8.41 |
| Q99720 | Sigma non-opioid intracellular receptor 1 | SIGMAR1 | 0 | 8 | 0 | 6 | 223 | 25.1 | 5.96 |
| Q15005 | Signal peptidase complex subunit 2 | SPCS2 | 1 | 4 | 0 | 5 | 226 | 25.0 | 8.47 |
| P51809 | Vesicle-associated membrane protein 7 | VAMP7 | 0 | 6 | 1 | 7 | 220 | 24.9 | 8.60 |
| O75396 | Vesicle-trafficking protein SEC22b | SEC22B | 0 | 8 | 0 | 6 | 215 | 24.6 | 6.92 |
| O43399-5 | Isoform 5 of Tumor protein D54 | TPD52L2 | 1 | 8 | 0 | 4 | 220 | 23.8 | 5.73 |
| O43402 | ER membrane protein complex subunit 8 | EMC8 | 0 | 9 | 0 | 17 | 210 | 23.8 | 6.40 |
| P51149 | Ras-related protein Rab-7a | RAB7A | 1 | 4 | 1 | 11 | 207 | 23.5 | 6.70 |
| P61026 | Ras-related protein Rab-10 | RAB10 | 1 | 8 | 1 | 11 | 200 | 22.5 | 8.38 |
| O15498 | Synaptobrevin homolog YKT6 | YKT6 | 1 | 8 | 1 | 4 | 198 | 22.4 | 6.92 |
| Q9H0U4 | Ras-related protein Rab-1B | RAB1B | 1 | 7 | 1 | 15 | 201 | 22.2 | 5.73 |
| Q8N4V1-2 | Isoform 2 of Membrane magnesium transporter 1 | MMGT1 | 2 | 9 | 1 | 8 | 196 | 21.9 | 9.54 |
| P84157 | Matrix-remodeling-associated protein 7 | MXRA7 | 0 | 8 | 1 | 5 | 204 | 21.5 | 4.26 |
| Q9P0T7 | Transmembrane protein 9 | TMEM9 | 0 | 4 | 0 | 4 | 183 | 20.6 | 6.65 |
| Q5J8M3 | ER membrane protein complex subunit 4 | EMC4 | 0 | 4 | 0 | 10 | 183 | 20.1 | 8.62 |
| O60831 | PRA1 family protein 2 | PRAF2 | 0 | 5 | 1 | 4 | 178 | 19.2 | 9.19 |
| P67812-4 | Isoform 4 of Signal peptidase complex catalytic subunit SEC11A | SEC11A | 1 | 7 | 0 | 5 | 164 | 19.0 | 8.40 |
| Q8WW12 | PEST proteolytic signal-containing nuclear protein | PCNP | 1 | 7 | 1 | 5 | 178 | 18.9 | 7.49 |
| P14209 | CD99 antigen | CD99 | 0 | 4 | 1 | 4 | 185 | 18.8 | 4.75 |
| P60604 | Ubiquitin-conjugating enzyme E2 G2 | UBE2G2 | 1 | 4 | 1 | 5 | 165 | 18.6 | 4.70 |
| Q6IAA8 | Ragulator complex protein LAMTOR1 | LAMTOR1 | 2 | 12 | 2 | 8 | 161 | 17.7 | 5.15 |
| Q9P003 | Protein cornichon homolog 4 | CNIH4 | 0 | 4 | 0 | 8 | 139 | 16.1 | 6.65 |
| Q8N5K1 | CDGSH iron-sulfur domain-containing protein 2 | CISD2 | 0 | 5 | 1 | 6 | 135 | 15.3 | 9.61 |
| P62987 | Ubiquitin-60S ribosomal protein L40 | UBA52 | 7 | 36 | 7 | 36 | 128 | 14.7 | 9.83 |
| Q9UHA4 | Ragulator complex protein LAMTOR3 | LAMTOR3 | 0 | 4 | 0 | 8 | 124 | 13.6 | 7.34 |
| Q9Y6A9 | Signal peptidase complex subunit 1 | SPCS1 | 1 | 5 | 0 | 5 | 102 | 11.8 | 9.31 |
| O75964 | ATP synthase subunit g | ATP5L | 1 | 4 | 0 | 4 | 103 | 11.4 | 9.64 |
