# Supplementary figures and images for: DRAM1 plays a tumor suppressor role in NSCLC cells by promoting lysosomal degradation of EGFR
Source: Cell Death Dis. 2020 Sep 17;11(9):768. doi: 10.1038/s41419-020-02979-9 (PMC7498585; doi:10.1038/s41419-020-02979-9)

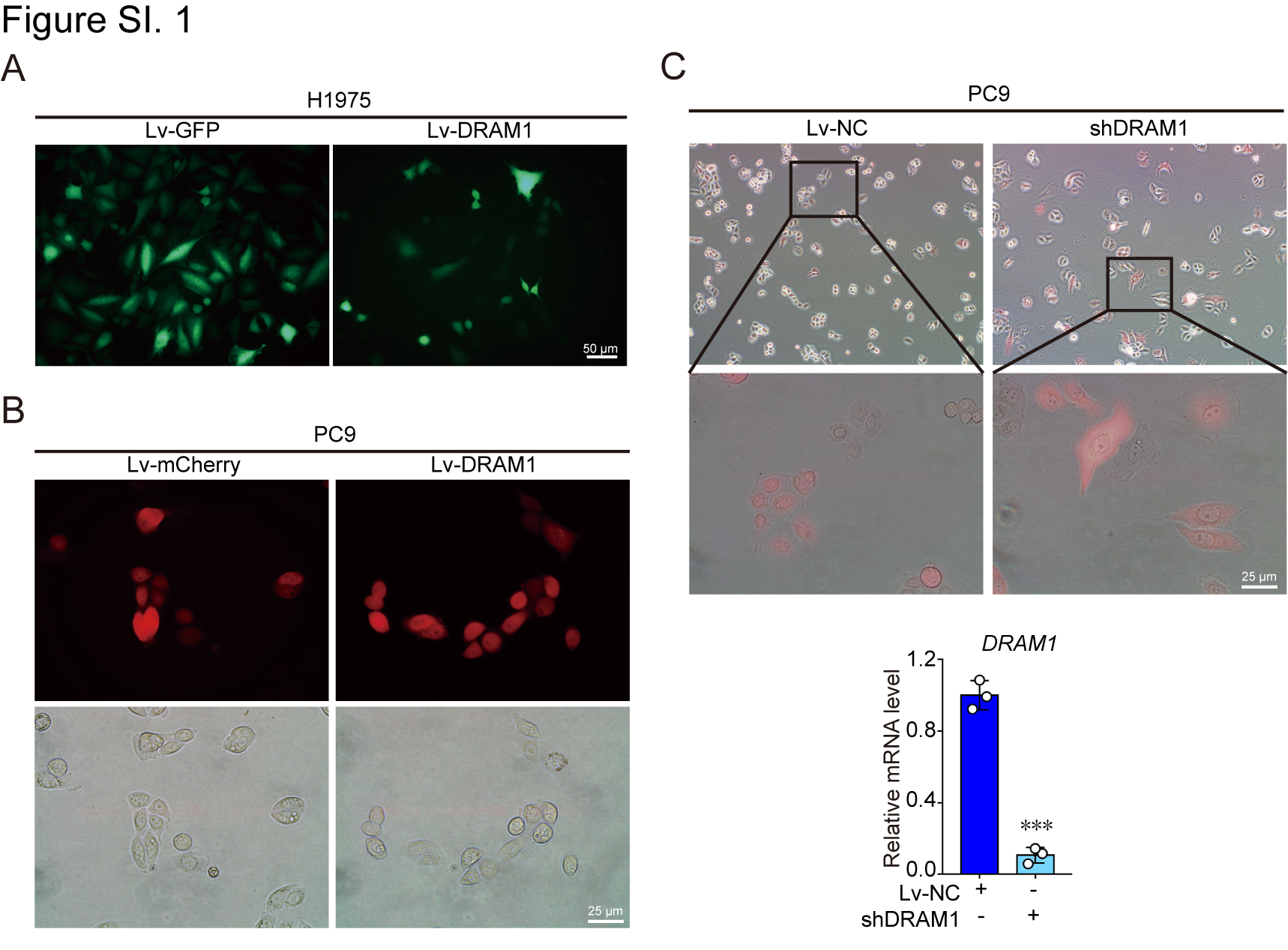

Supplement: Supplementary file 5 — Figure SI 1 [file 41419_2020_2979_MOESM5_ESM.tif]

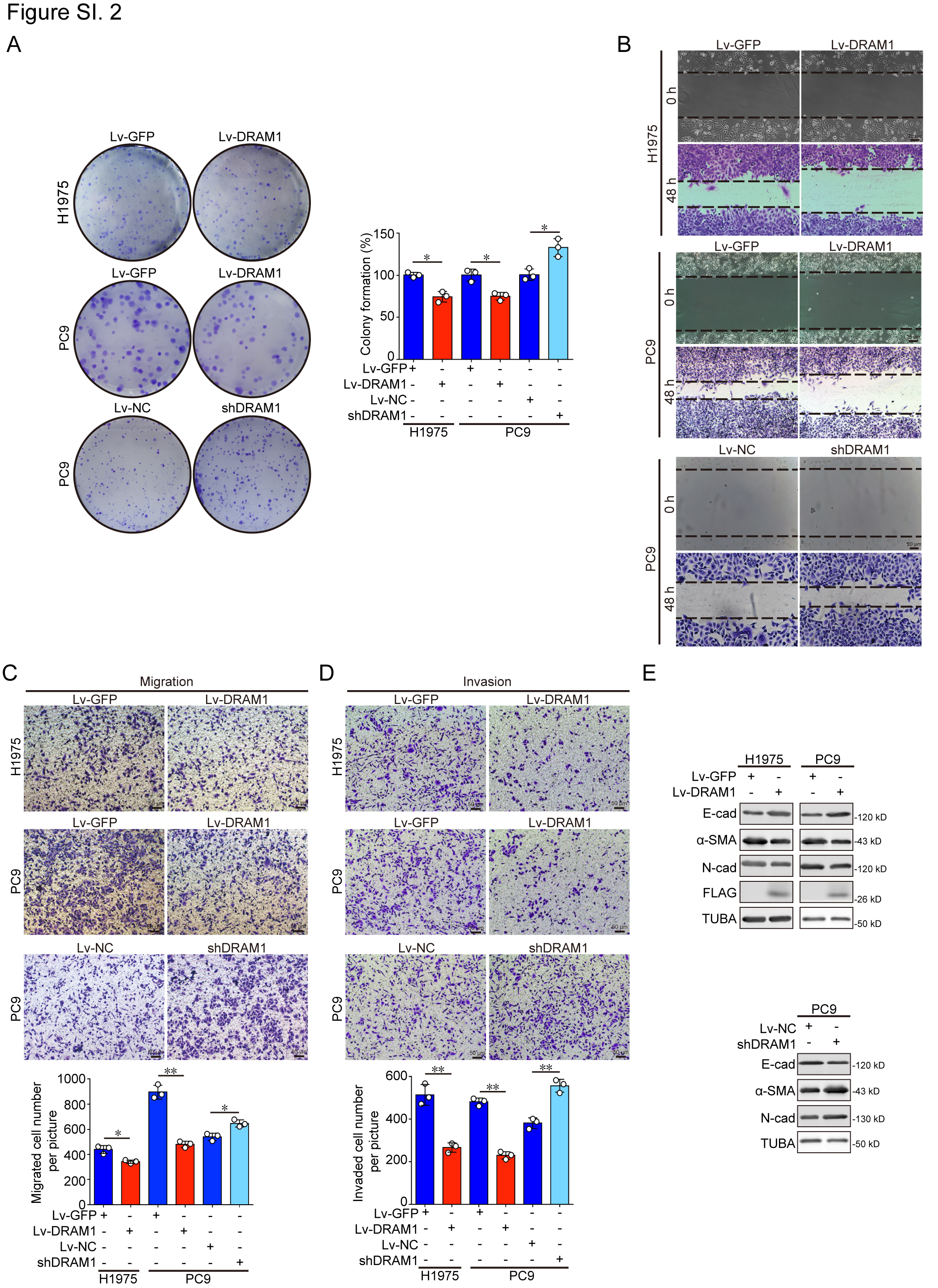

Supplement: Supplementary file 6 — Figure SI 2 [file 41419_2020_2979_MOESM6_ESM.tif]

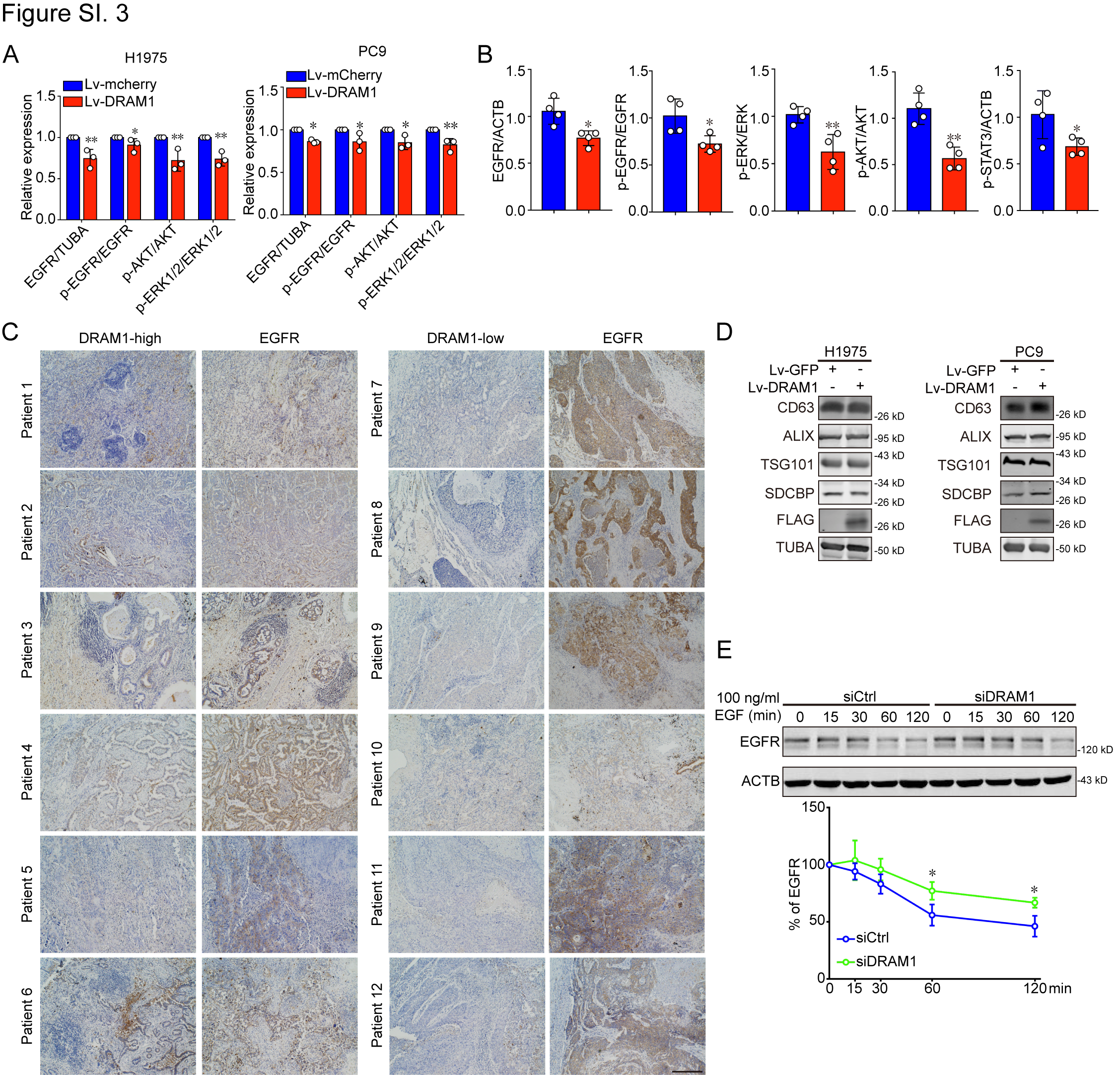

Supplement: Supplementary file 7 — Figure SI 3 [file 41419_2020_2979_MOESM7_ESM.tif]

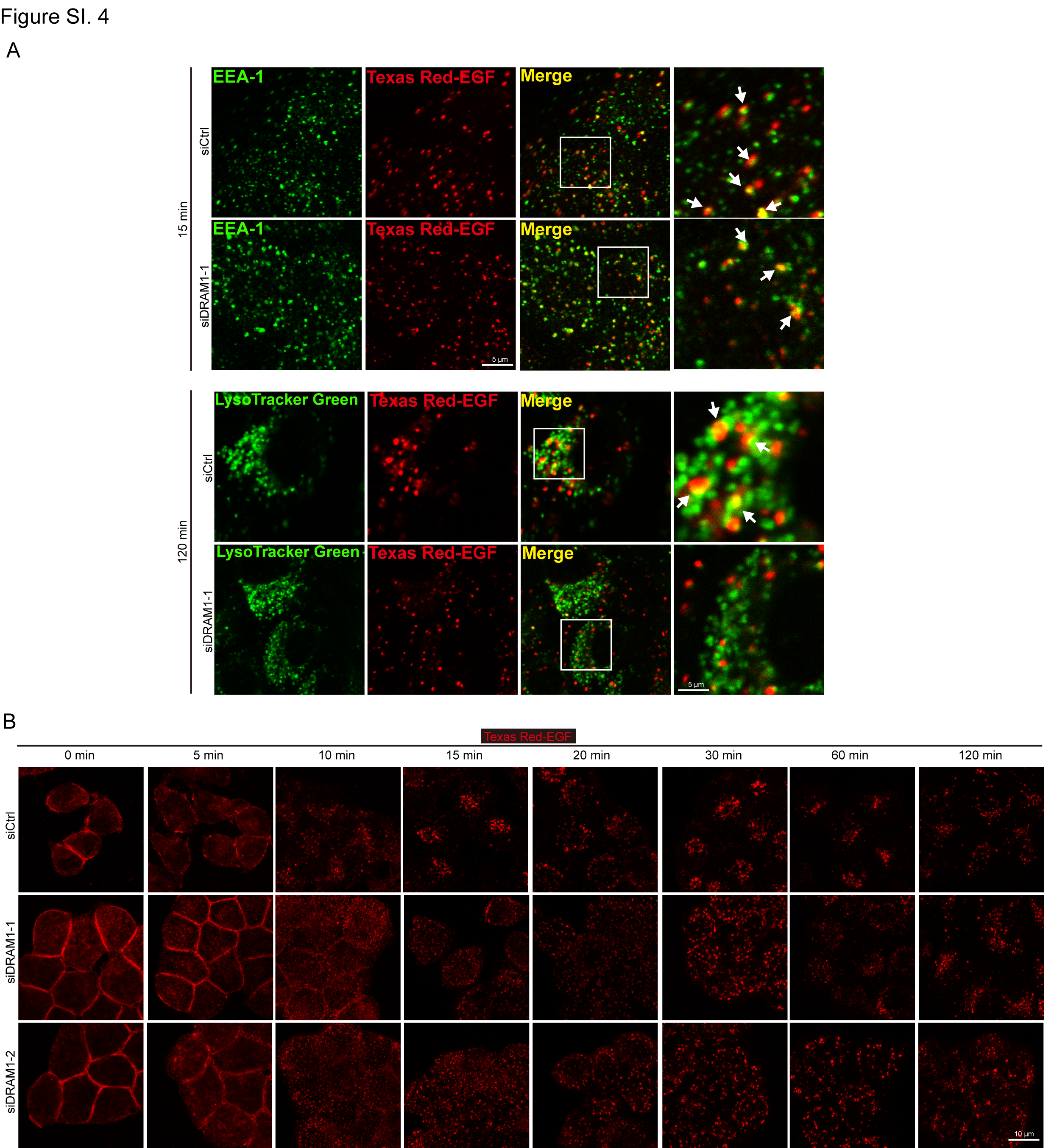

Supplement: Supplementary file 8 — Figure SI 4 [file 41419_2020_2979_MOESM8_ESM.tif]

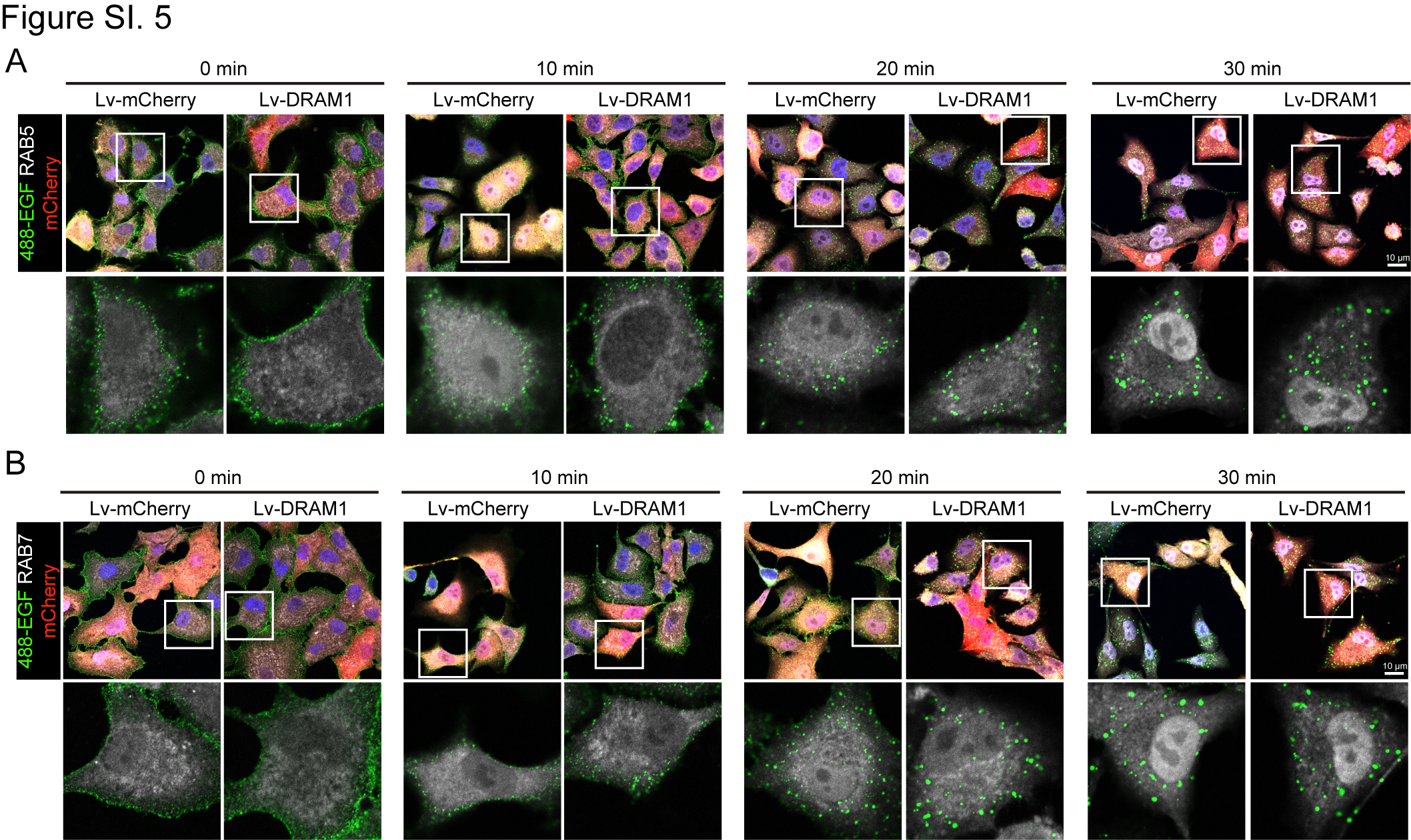

Supplement: Supplementary file 9 — Figure SI 5 [file 41419_2020_2979_MOESM9_ESM.tif]

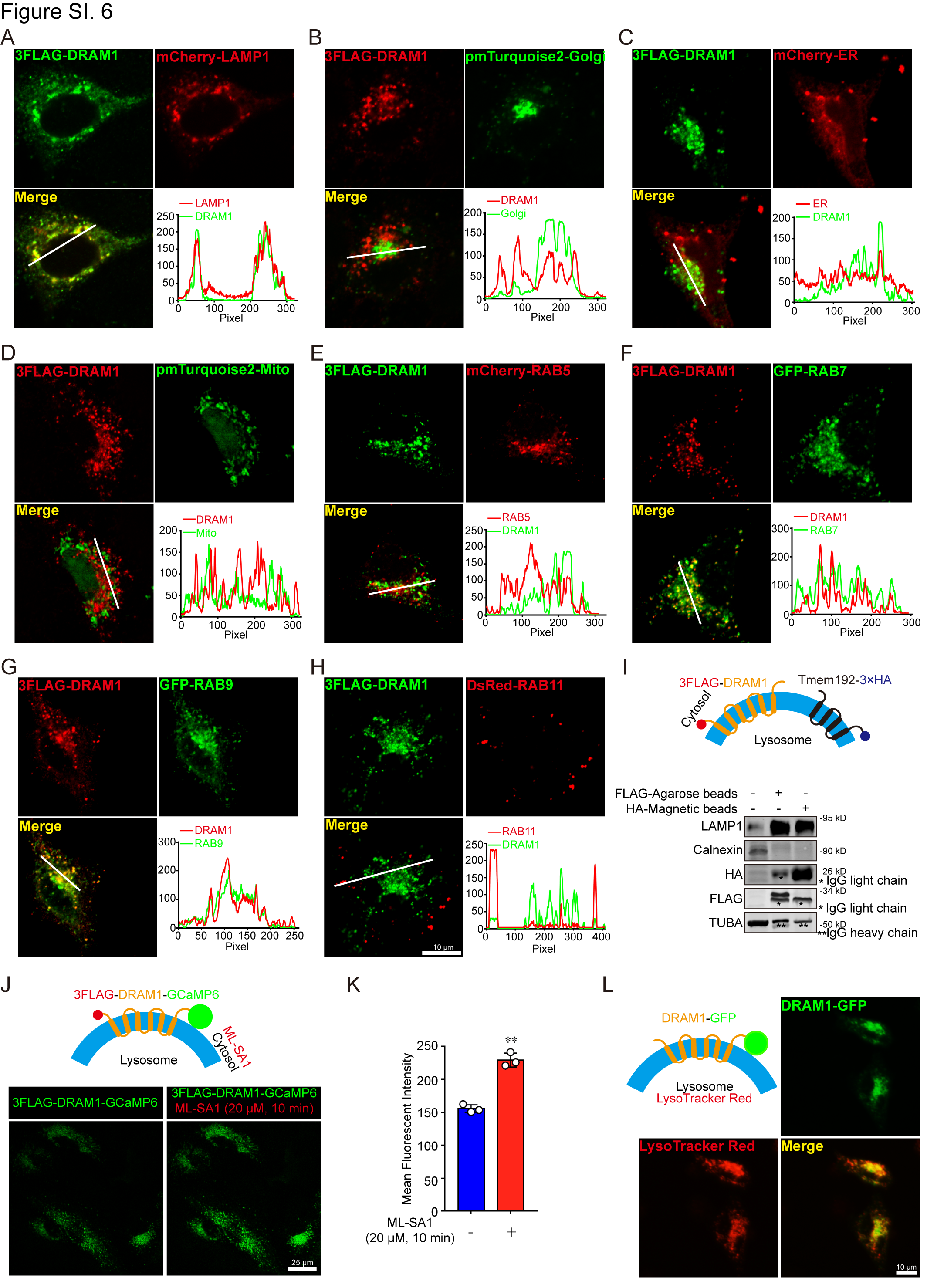

Supplement: Supplementary file 10 — Figure SI 6 [file 41419_2020_2979_MOESM10_ESM.tif]

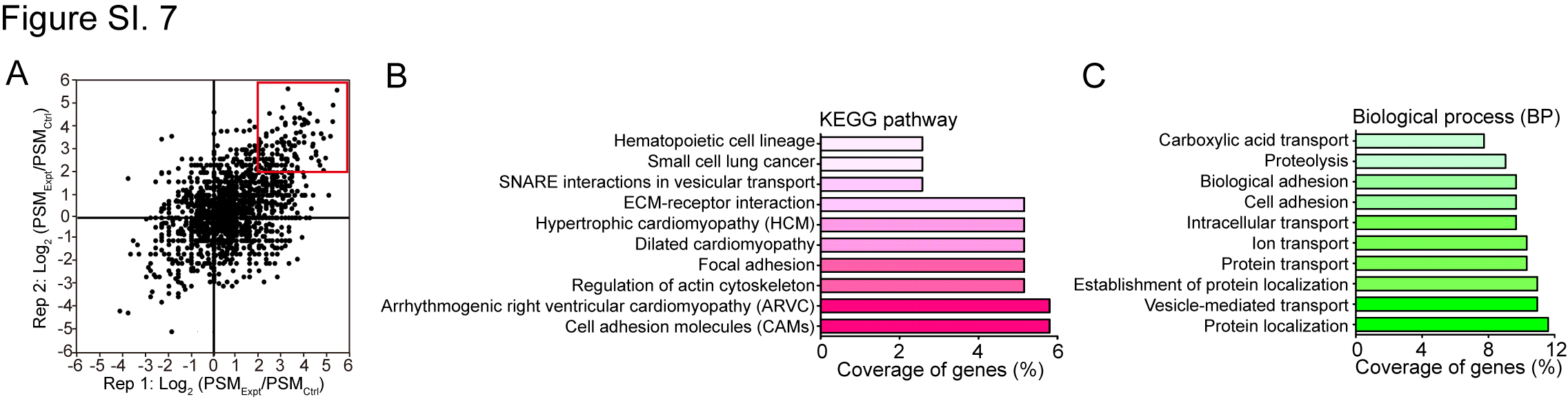

Supplement: Supplementary file 11 — Figure SI 7 [file 41419_2020_2979_MOESM11_ESM.tif]

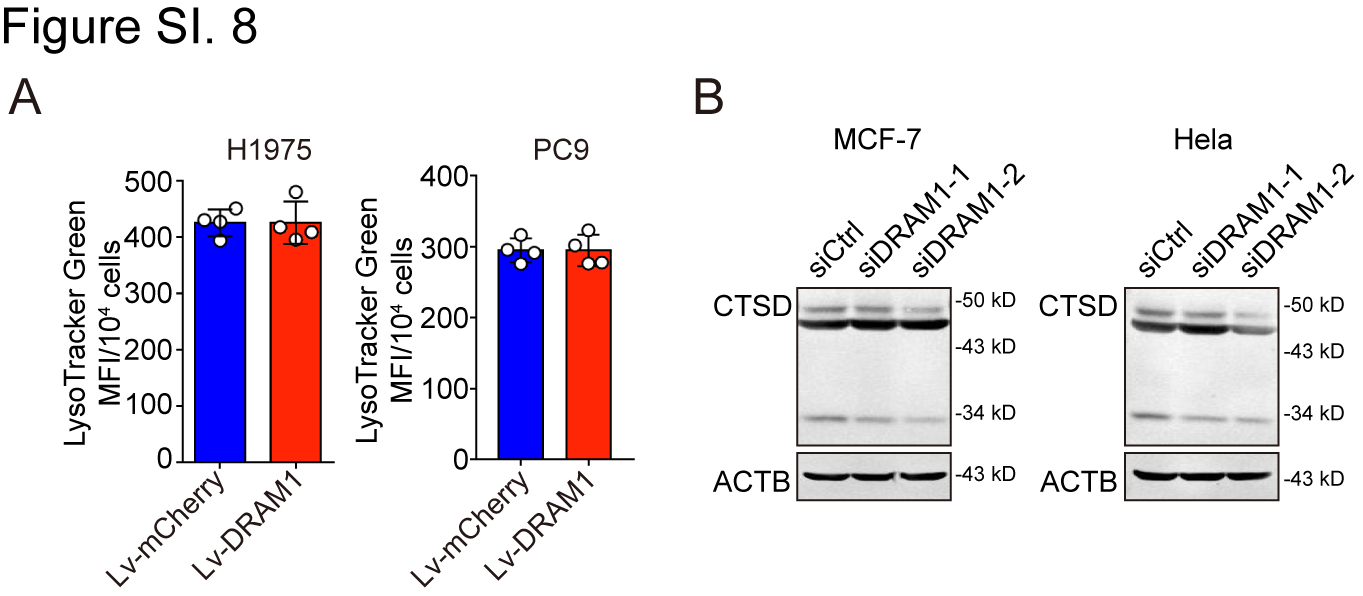

Supplement: Supplementary file 12 — Figure SI 8 [file 41419_2020_2979_MOESM12_ESM.tif]

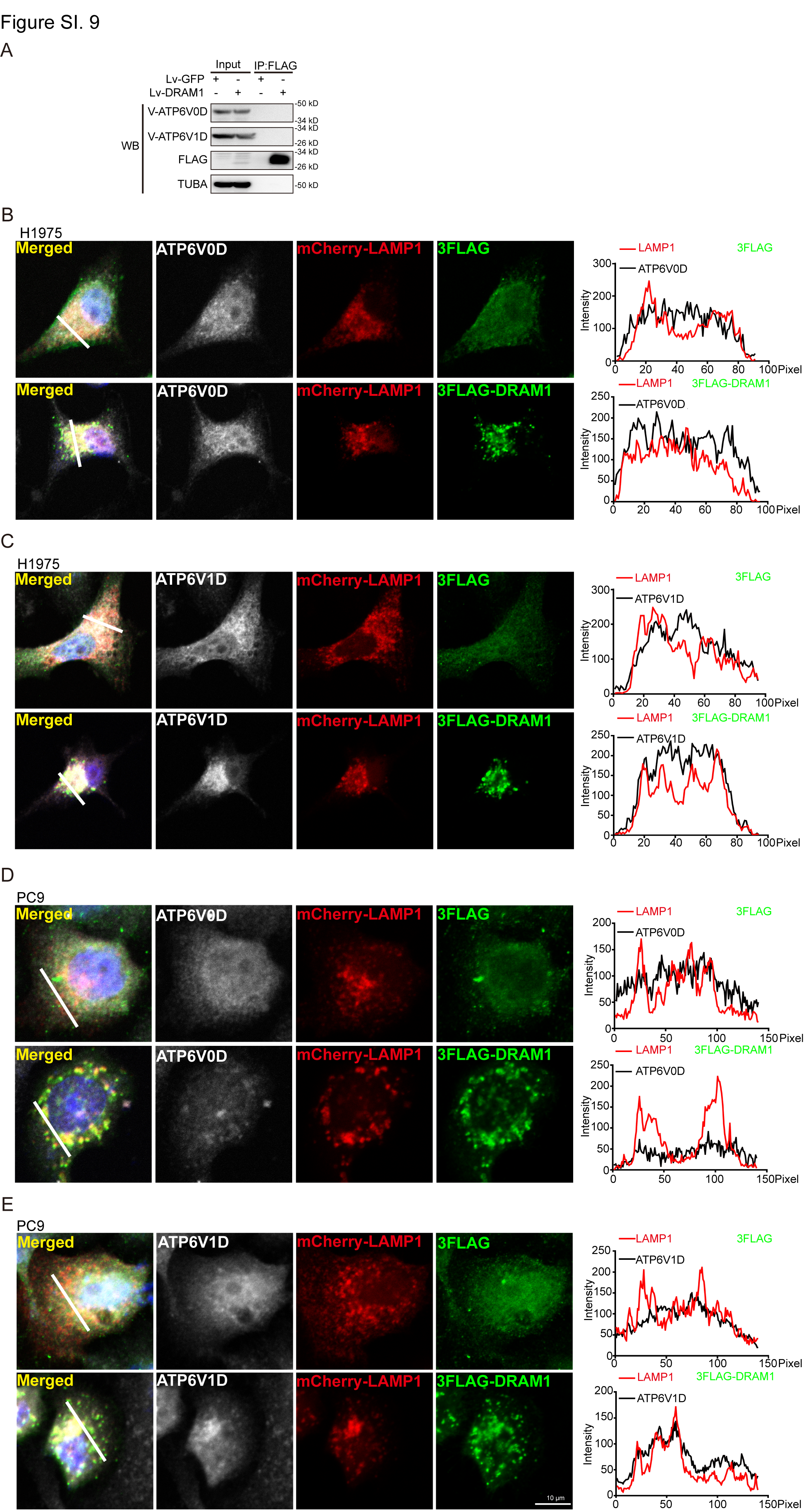

Supplement: Supplementary file 13 — Figure SI 9 [file 41419_2020_2979_MOESM13_ESM.tif]

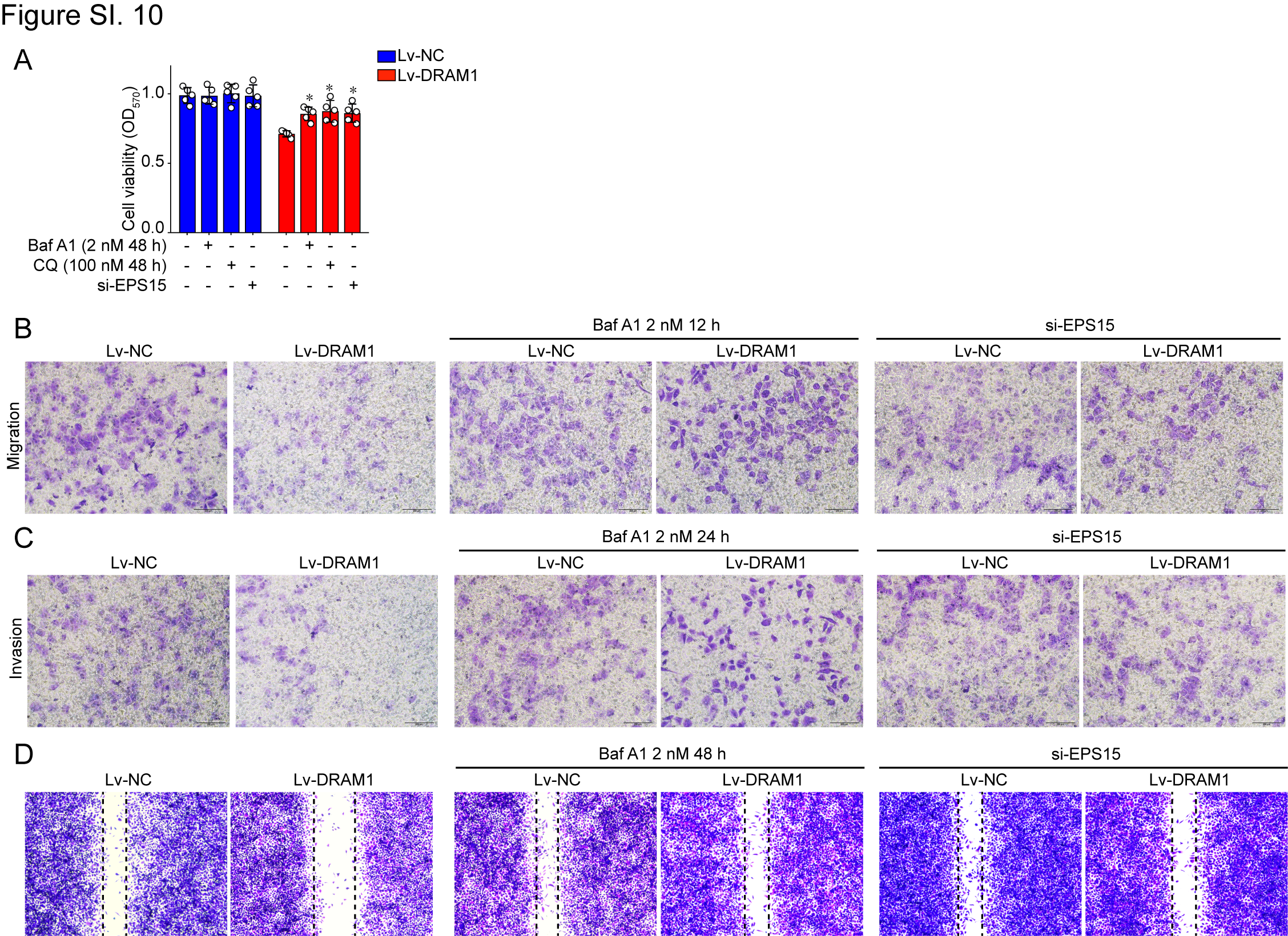

Supplement: Supplementary file 14 — Figure SI 10 [file 41419_2020_2979_MOESM14_ESM.tif]
